# A computational strategy for the search of regulatory small RNAs in *Actinobacillus pleuropneumoniae*

**DOI:** 10.1261/rna.055129.115

**Published:** 2016-09

**Authors:** Ciro C. Rossi, Janine T. Bossé, Yanwen Li, Adam A. Witney, Kate A. Gould, Paul R. Langford, Denise M.S. Bazzolli

**Affiliations:** 1 Laboratório de Genética Molecular de Micro-organismos, Departamento de Microbiologia, Instituto de Biotecnologia Aplicada à Agropecuária–BIOAGRO, Universidade Federal de Viçosa, Viçosa, 36570-900, Brazil; 2 Section of Paediatrics, Imperial College London, St. Mary's Campus, London W2 1PG, United Kingdom; 3 Institute for Infection and Immunity, St. George's, University of London, London SW17 0RE, United Kingdom

**Keywords:** sRNAs, *Pasteurellaceae*, porcine pleuropneumonia, bioinformatics, virulence

## Abstract

Bacterial regulatory small RNAs (sRNAs) play important roles in gene regulation and are frequently connected to the expression of virulence factors in diverse bacteria. Only a few sRNAs have been described for *Pasteurellaceae* pathogens and no in-depth analysis of sRNAs has been described for *Actinobacillus pleuropneumoniae*, the causative agent of porcine pleuropneumonia, responsible for considerable losses in the swine industry. To search for sRNAs in *A. pleuropneumoniae*, we developed a strategy for the computational analysis of the bacterial genome by using four algorithms with different approaches, followed by experimental validation. The coding strand and expression of 17 out of 23 RNA candidates were confirmed by Northern blotting, RT-PCR, and RNA sequencing. Among them, two are likely riboswitches, three are housekeeping regulatory RNAs, two are the widely studied GcvB and 6S sRNAs, and 10 are putative novel *trans*-acting sRNAs, never before described for any bacteria. The latter group has several potential mRNA targets, many of which are involved with virulence, stress resistance, or metabolism, and connect the sRNAs in a complex gene regulatory network. The sRNAs identified are well conserved among the *Pasteurellaceae* that are evolutionarily closer to *A. pleuropneumoniae* and/or share the same host. Our results show that the combination of newly developed computational programs can be successfully utilized for the discovery of novel sRNAs and indicate an intricate system of gene regulation through sRNAs in *A. pleuropneumoniae* and in other *Pasteurellaceae*, thus providing clues for novel aspects of virulence that will be explored in further studies.

## INTRODUCTION

Bacterial regulatory RNAs represent a diverse class of regulators that operate at all layers of gene regulation, ranging from transcriptional initiation to protein translation (Papenfort and Vogel 2010; Harris et al. 2013). An emerging class of such regulators is from 40–500 nucleotides (nt) in length and is thus called small RNAs—sRNAs (Li et al. 2012).

Most sRNAs can be divided into the following four broad categories: (i) *cis*-acting RNAs; *trans*-acting RNAs that may either (ii) modulate protein activity or (iii) bind to mRNAs; and (iv) clustered regularly interspaced short palindromic repeats—CRISPRs (Michaux et al. 2014b). The most studied bacterial sRNAs are the ones coded in *trans*, which exert their cellular roles by base pairing with mRNA targets to attenuate, stop, or activate their translation (Man et al. 2011; Papenfort and Vanderpool 2015). These sRNAs normally have more than one target since they only have limited complementarity with their cognate mRNAs (Han et al. 2013). Because of this partial complementarity, some of them may rely on the molecular chaperone Hfq to mediate their proper interaction with the cognate mRNAs by remodeling and stabilizing their structure, in addition to stimulating annealing (Vogel and Luisi 2011). sRNAs that interact with proteins include the 6S RNA, which binds to the primary holoenzyme form of RNA polymerase and affects the expression of housekeeping genes under low nutrient conditions (Cavanagh and Wassarman 2014), and the sRNA CsrB, which is the major regulator of the protein CsrA—the effector of the complex network of the carbon storage regulatory (Csr) system controlling various virulence-related and metabolic phenotypes in several bacteria (Vakulskas et al. 2015).

Among the regulatory RNAs that act in *cis*, antisense sRNAs and riboswitches are the most important. Antisense sRNAs are transcribed from the DNA strand opposite their target gene on the bacterial chromosome, with which they have perfect complementarity (Thomason and Storz 2010). These RNAs can also be regulators of virulence and stress response in important pathogens (Gomez-Lozano et al. 2014b; Cho and Kim 2015). Riboswitches consist of mRNAs’ regulatory segments, which alter their conformation in response to the presence of a particular metabolite, usually causing the ribosome binding site in the cognate mRNA to be blocked or exposed (Mandal and Breaker 2004; Narberhaus et al. 2006).

CRISPR elements and the CRISPR-associated (Cas) proteins are considered the adaptive immunity system in prokaryotes that function via a mechanism of foreign DNA fragment (mainly bacteriophages and plasmids) incorporation into repeated arrays and subsequent utilization of transcripts of these inserts (known as spacers) as guide RNAs to cleave the cognate selfish element genome (Koonin and Wolf 2015).

Since sRNAs play versatile roles in the bacterial cell, a determined sRNA profile guarantees a quick and precise process of gene regulation and physiological adaptation to an ever-changing environment, which may be necessary for the establishment of a bacterial pathogenic lifestyle (Michaux et al. 2014b).

Many studies of novel sRNA identification rely on RNA sequencing (Li et al. 2013; Bilusic et al. 2014; Gomez-Lozano et al. 2014a), but because most sRNAs in bacterial transcriptomes correspond to a few overexpressed structural RNAs or products of mRNA degradation, only a portion of these RNAs is identified in laboratory approaches, requiring more expensive and time-consuming protocol adaptations (Gomez-Lozano et al. 2014a). For that reason, computational tools have become relevant, with ever-growing approaches for the discovery and characterization of regulatory RNAs (Cros et al. 2011; Livny 2012; Tesorero et al. 2013; Corredor and Murillo 2014).

In this work, we focused on the pathogenic bacterium *Actinobacillus pleuropneumoniae*, the causative agent of swine pleuropneumonia, a severe necrotic, fibrinous, and hemorrhagic disease (Bossé et al. 2002; Krejci and Newberry 2011). *A. pleuropneumoniae* is a Gram-negative microaerophilic coccobacillus of the family *Pasteurellaceae*. The pathogenesis of pleuropneumonia is complex and involves many virulence factors, of which the Apx toxins, of the RTX family, are believed to play a central role (Frey 2011). Recently, *A. pleuropneumoniae hfq* mutants were shown to be defective in biofilm formation, displayed enhanced sensitivity to oxidative stress (Subashchandrabose et al. 2013), and were attenuated in an alternative infection model (Pereira et al. 2015b). Hfq is an RNA-binding protein that facilitates the pairing of sRNAs with their target mRNAs and affects gene expression (Vogel and Luisi 2011). Although these findings indicate that *A. pleuropneumoniae* may rely on sRNAs to control aspects of its virulence, no studies specifically addressing the identification and potential role of regulatory RNAs have been reported for this bacterium so far. In addition, little is known about the role of sRNAs in other *Pasteurellaceae*. The first study of these regulators in this family was performed on the human oral pathogen *Aggregatibacter actinomycetemcomitans*, in which three novel iron-regulated sRNAs were identified (Amarasinghe et al. 2012), followed by the discovery of the iron-regulated HffR sRNA in *Haemophilus influenzae* (Santana et al. 2014). Thus, the main goal of this study was to establish a strategy using free, web-accessible and user-friendly computational tools for the identification of regulatory RNAs in *A. pleuropneumoniae* and other bacteria from the same family. Selected examples of predicted sRNAs were confirmed by Northern blotting and/or RT-PCR demonstrating the utility of the approach.

## RESULTS

### Computational prediction of regulatory RNAs in *A. pleuropneumoniae*

**FIGURE 1.**
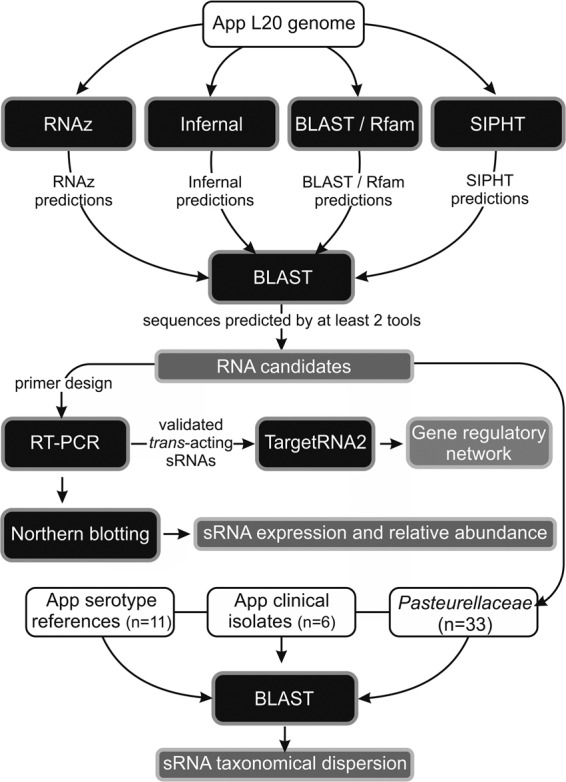
A strategy for the search and characterization of regulatory RNAs in *A. pleuropneumoniae* (App). Black rectangles represent the tools, both computational and laboratorial, for the prediction, validation, and characterization of noncoding regulatory RNA in *A. pleuropneumoniae*. White rectangles show inputs and gray rectangles show the outputs (and final objectives) in the workflow, represented by the arrows.

For the discovery of novel regulatory RNAs in the *A. pleuropneumoniae* L20 genome, we used a computational strategy relying on a combination of four algorithms each using a different approach, i.e., searching across the whole genome for evolutionarily conserved and thermodynamically stable secondary structures (RNAz), intergenic regions that may contain transcriptional terminators (SIPHT), or may form stable secondary structures or characteristic motifs (INFERNAL), in addition to previously described sRNAs (BLASTn against Rfam), followed by experimental validation (Fig. 1). Because each program can generate a high number of candidates, the results obtained from each method were compared with one another to increase prediction accuracy. Sequences that were predicted by at least two different algorithms were considered to be sRNA candidates for further evaluation.

**FIGURE 2.**
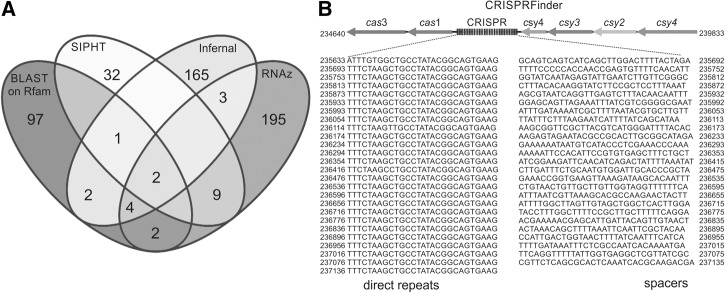
Computational prediction of small regulatory RNAs in *A. pleuropneumoniae*. (*A*) The sRNA candidates were defined as those predicted by at least two out of four (BLAST/Rfam, SIPHT, Infernal, and RNAz) algorithms with different approaches. As shown by the Venn diagram's intersections, the number of final candidates selected adopting this criterion was 23. (*B*) A CRISPR locus was also searched by using the software CRISPRFinder. For the *A. pleuropneumoniae* reference strain L20, a total of 26 direct repeats, separated by 25 spacers (gray) and surrounded by CRISPR-associated genes (arrows), were found.

The algorithms used (RNAz, INFERNAL, SIPHT, and BLASTn against Rfam) predicted 215, 177, 44, and 108 genomic segments as putative regulatory RNAs, respectively. Many RNAs predicted by BLASTn/Rfam were tRNAs or rRNAs and were discarded from the analysis. The final number of RNA candidates considered after checking the intersection of the four results was 23, as shown by the Venn diagram in Figure 2A. They were named as Arrc01-23, from ***A****ctinobacillus pleuropneumoniae* **R**egulatory **R**NA **C**andidate.

Among the 23 candidates, eight (Arrc01, 03, 06, 10, 13, 15, 17, and 19) were predicted by at least three algorithms, with Arrc01 and Arrc15 predicted by all four used. All the Arrc loci are within intergenic regions and thus are not annotated in the *A. pleuropneumoniae* L20 genome, or any of the other complete genomes from this species available in the public databases. They are described in Table 1.

**TABLE 1.**
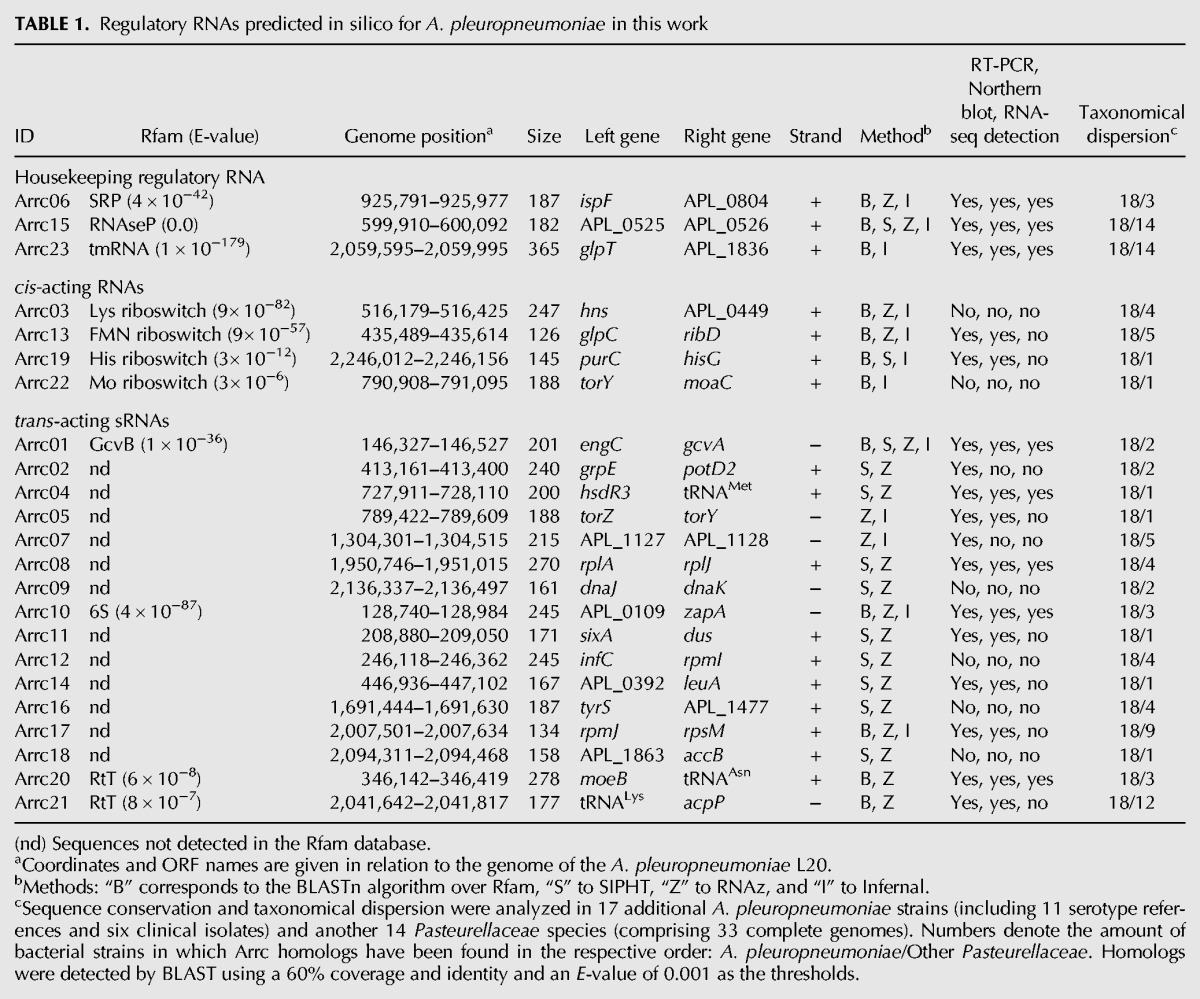
Regulatory RNAs predicted in silico for *A. pleuropneumoniae* in this work

The RNA candidates identified can be classified in different categories, such as housekeeping regulatory RNAs, *trans*-acting sRNAs modulating protein activity, *trans*-acting sRNAs regulating mRNAs, and *cis*-acting RNAs, as will be presented in the next section. The present strategy was not designed to detect CRISPRs, as these are not targets of the algorithms used. However, a separate search with the CRISPRFinder program (Grissa et al. 2007) detected a CRISPR element of 1503 bases, composed of 26 typical nearly identical sequences of 28 bases each, all separated by spacers (25 in total) that are on average 32 bases long and surrounded by *cas* genes (Fig. 2B).

### Verification of the regulatory RNA candidates’ expression

**FIGURE 3.**
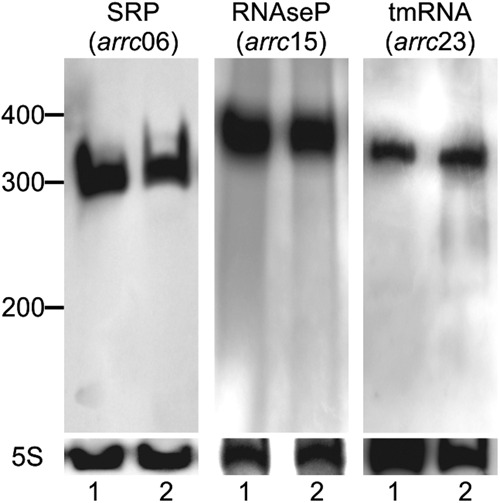
Housekeeping regulatory RNAs predicted by bioinformatics for *A. pleuropneumoniae* L20. Expression was validated by Northern blotting under aerobic (1) and anaerobic (2) conditions.

**FIGURE 4.**
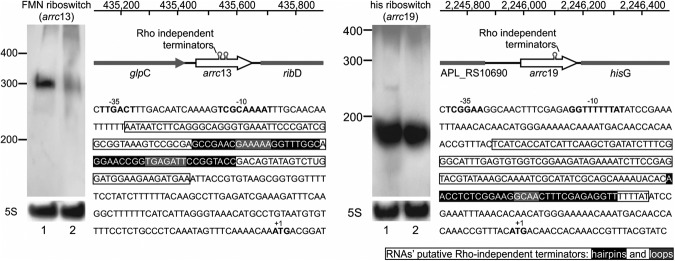
*Cis*-acting regulatory RNAs predicted for *A. pleuropneumoniae*. Expression was validated by Northern blotting under aerobic (1) and anaerobic (2) conditions. The genomic context, promoter region, putative terminators, and controlled gene translation starting point are shown for *A*. *pleuropneumoniae* L20.

**FIGURE 5.**
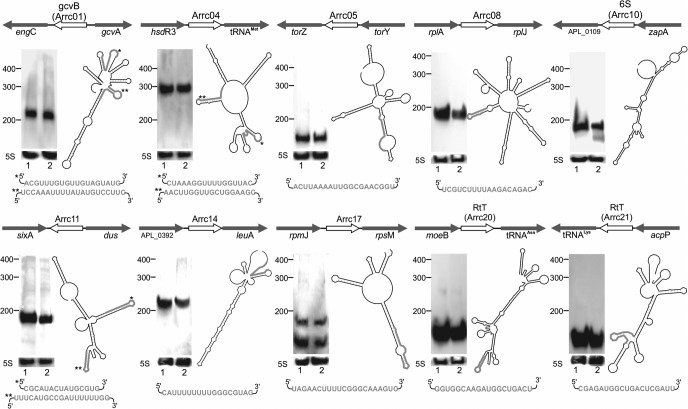
Putative *trans*-acting sRNAs predicted for *A. pleuropneumoniae*. Expression was validated by Northern blotting under aerobic (1) and anaerobic (2) conditions. The predicted secondary structures show the formation of several hairpin regions, defined by the presence of palindromic sequences. Target prediction with TargetRNA2 reveals preferable binding sites (except for RNA polymerase-interacting 6S—Arrc10 sRNA), highlighted in gray. Genomic context is shown for the *A. pleuropneumoniae* reference strain L20.

Prior to performing Northern blotting, the coding strand and expression of the sRNA candidates was verified by RT-PCR. The coding strand was determined by using only the forward or the reverse primer, designed for each candidate, in the cDNA synthesis reaction. By doing so, only the reaction to which the primer capable of annealing to the sRNA was added would generate a cDNA product to be detected in the next PCR step of the protocol. In this analysis, we detected the expression of 17 of the 23 (74%) Arrcs (data not shown). It was not possible to detect the expression of Arrc03, 09, 12, 16, 18, and 22 in the conditions evaluated. Then, to confirm the expression and relative abundance of the candidates, Northern blotting was used. In every case, the Northern blot showed a discrete band with a size similar to the predicted sRNA, and in some situations, the blot also showed additional bands. The *A*. *pleuropneumoniae* ribosomal small RNA 5S was used as a positive control (Figs. 3–5).

#### Housekeeping regulatory RNAs

Although more than half of all the RNAs predicted herein have not previously been described, the identity of some could be inferred by homology searches (BLASTn) against the main public databases. For example, Arrc06 is a widely distributed housekeeping RNA that is the functional RNA component of the signal recognition particle (SRP) that delivers nascent peptides to their proper destination (Grotwinkel et al. 2014). Also very conserved and widespread, but with activities not related to the interaction with proteins, are the RNAs Arrc15 and Arrc23. Arrc15 is the ribozyme RNAseP, involved in processing tRNAs (Evans et al. 2006), and Arrc23 is a tmRNA, with dual tRNA-like and mRNA properties, which plays a central role in the process of recycling ribosomes stalled in aberrant mRNAs (Keiler and Ramadoss 2011). Both RNAseP and tmRNA use protein cofactors, which are also present in the *A. pleuropneumoniae* genome. The genes *smpB* (small protein B, cofactor of tmRNA) and *rnpA* (protein C5, cofactor of RNAseP) are in the following position of the *A. pleuropneumoniae* L20 genome sequence, respectively: 1,006,549–1,007,028 and 2,172,077–2,172,379. As would be expected, the expression of all of these RNAs was observed by Northern blotting (Fig. 3). No apparent differences in their level of expression were observed between the aerobic and anaerobic growth conditions.

#### *Cis*-acting RNAs

Our approach also predicted four *cis*-acting regulatory RNAs. Arrc03, Arrc13, Arrc19, and Arrc22 are homologous to the lysine, flavine mononucleotide (FMN), histidine, and molybdenum riboswitches, respectively. These annotations are consistent with the genome localization of the candidates Arrc13, Arrc19, and Arrc22, as the first one is upstream of the gene *ribD* (riboflavin biosynthesis protein), the second is upstream of the gene *hisG* (an ATP phosphoribosyl transferase involved in histidine biosynthesis), and the third is upstream of the gene *moaA* (molybdenum cofactor biosynthesis protein A). The annotation of Arrc03, however, remains unclear, since in its vicinity are the gene *hns* and that for a hypothetical protein which by BLASTn belongs to a family of Na^+^/H^+^ anti-porters (Pfam ID: pfam03553), not directly related to lysine biosynthesis, as expected.

Only the expression of the FMN and *his* riboswitches were observed by both RT-PCR and Northern blotting (Fig. 4). As expected for producing a coenzyme of the electron respiratory chain, the expression of FMN was more prominent during aerobic growth, while no apparent difference could be observed in the expression of the histidine riboswitch—probably because no nutritional stress was implicated in the growth conditions tested. These sRNA structures are in agreement with the fact that *cis*-regulatory elements usually include intrinsic attenuators (secondary structures shown in Supplemental Fig. S1), frequently formed upon binding of the target molecule, thereby prematurely terminating transcription. Because riboswitches can be regulators at the transcription level (Henkin 2008), and the RNA extraction protocol was specific for purifying small molecules, the Northern blots of Arrc13 and Arrc19 showed a specific small band for the *cis*-element alone, instead of the entire unit containing the riboswitch and the regulated mRNA.

#### GcvB, 6S, and other putative trans-acting sRNAs

Among the 12 remaining regulatory RNAs whose expression was detected by RT-PCR, two are well-studied sRNAs, and 10 are likely novel *trans*-acting RNAs. Arrc01 is the vastly studied GcvB, a major regulator of amino acid metabolism (Stauffer and Stauffer 2013), and Arrc10 is the global transcription regulator 6S RNA. Additionally, both Arrc20 and Arrc21 are homologous to sRNAs with broader targets. In the Rfam database, they belong to the RtT family, which in *Escherichia coli* was discovered as a RNA molecule liberated from the transcript of a tRNA operon and was implicated in cellular responses to face amino acid limitations in the cell (Bosl and Kersten 1991). Their annotation is consistent with their location upstream of tRNA genes. Arrc20 is upstream of an Asn-tRNA gene and Arrc21 begins upstream of a Lys-tRNA gene.

With the exception of the Arrcs 02 and 07, all the other putative sRNAs showed consistent signals on Northern blots, including GcvB, the 6S and the RtTs (Fig. 5). While most sRNAs displayed single and specific bands, Arrc10 and Arrc17 exhibited additional shorter bands. With the exception of the additional shorter band observed for Arrc10 when the bacterium was grown in anaerobiosis, no other evident differences in any of the *trans* sRNAs could be observed when the bacterium was grown aerobically or anaerobically. Because of the abundance in palindromic sequences—which aided in their discovery—all the sRNAs are possibly able to form complex secondary structures composed of several hairpins, as predicted by RNAfold (Gruber et al. 2008).

#### RNA sequencing

The expression of the regulatory RNA candidates was also investigated by RNA sequencing (RNA-seq) after bacterial growth under aerobic and anaerobic conditions. Because the RNA-seq experiments resulted in a low number of reads (1,106,169 for aerobic and 1,333,114 for anaerobic growth) and no differential expression (*P* < 0.05) between the two conditions was observed by Cuffdiff (Trapnell et al. 2012)—data not shown—the search for our RNA candidates was performed in an assembled transcriptome built after merging the aerobic and anaerobic reads. The resulting mapping files generated were uploaded to NCBI's SRA (Sequence Read Archive) under the experiment SRX810211. From that transcriptome we could confirm the expression of the RNAs Arrc01, 04, 06, 08, 10, 15, 20, 21, and 23.

Overall, from 23 predicted RNAs in *A. pleuropneumoniae*, the expression of 17 was confirmed under the conditions tested. From these, three are housekeeping regulatory RNAs (SRP—Arrc06, RNAseP—Arrc15, and tmRNA—Arrc23), two are *cis*-acting RNAs (FMN—Arrc13 and *his*—Arrc19 riboswitches), two are well-known *trans*-acting sRNAs (GcvB—Arrc01 and 6S—Arrc10), ten are putative *trans*-acting sRNAs, of which two have Rfam homologues (RtTs—Arrc20 and Arrc21) and eight are novel sRNAs (Arrc02, 04, 05, 07, 08, 11, 14, and 17). All the candidates had putative Rho-independent terminator regions and promoter elements in the close upstream region of each designated gene, as predicted by BPROM (software Softberry, available at www.softberry.com, Supplemental Fig. S2). None of the sRNA genes described in this article have previously been annotated in the publicly available *A. pleuropneumoniae* genomes and represent, thus, an expansion in the understanding of the genome content of this microorganism.

### The *trans*-acting sRNAs are potentially involved in an intricate network of gene regulation

To investigate the possible targets and roles of the aforementioned validated *trans*-acting sRNAs (Arrc01, 02, 04, 05, 07, 08, 11, 14, 17, 20, and 21), we performed a computational target prediction with TargetRNA2 (Kery et al. 2014), considering all the annotated ORFs in the *A. pleuropneumoniae* L20 genome. The interactions within the vicinity of the mRNA translational start site with the lowest energies and *P*-value below 0.05 were considered to indicate the best mRNA candidates. These targets are depicted in Supplemental Table S2. Following the search criteria established, TargetRNA2 predicted from 7 (for Arrc20) to 36 (for Arrc02)—with an average of 19.09 ± 8.53—known genes whose mRNAs present a great probability of binding the respective sRNA. Most of these targets are predicted to preferentially bind to conserved specific regions of the cognate sRNA (Fig. 5).

**FIGURE 6.**
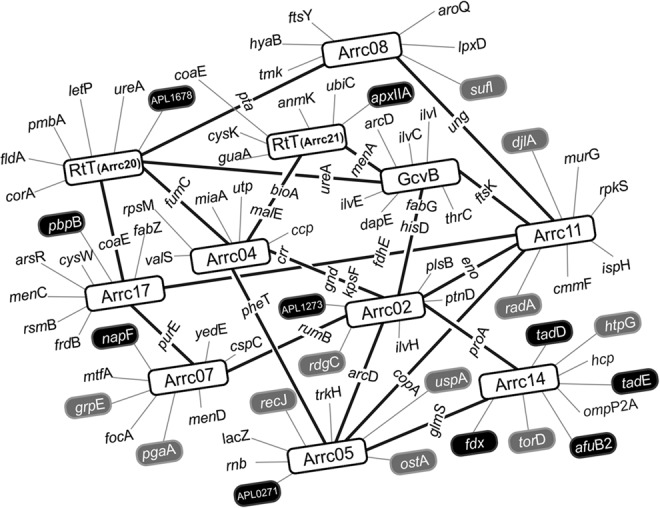
Regulatory network formed by *trans*-acting sRNAs and cognate mRNAs in *A. pleuropneumoniae.* The novel sRNAs described in this work have the potential to bind several mRNA targets, many of which are shared by more than one sRNA, possibly configuring an entangled network of gene regulation in *A. pleuropneumoniae*. The sRNA candidates’ names are depicted in white rectangles (Arrcs) and their mRNA connections are illustrated by thick black lines. The other putatively exclusive gene targets are linked to their respective regulatory RNA by gray lines. Targets that may be involved with either virulence or stress resistance are highlighted in black and gray, respectively.

Because some of the target genes are common to more than one sRNA, many sRNAs are likely linked in an entangled and complex gene regulatory network (Fig. 6). Many candidates have the potential to control the translation of mRNAs directly involved in virulence. For example, the mRNA for the gene *apxIIA*, encoding one of the Apx exotoxins, is predicted to be one of the targets of Arrc21 (RtT). Several sRNAs potentially bind mRNAs from genes whose products are involved in the intake and transport of iron from the host. Arrc05 may bind the RNA from the locus APL_0271, whose product is part of an iron transport system. Likewise, Arrc14 may control the expression of a ferric permease (gene *afuB2*). The expression of different ferredoxins, encoded by the genes *napF*, *fdx*, and locus APL_1678 are also potentially controlled by the sRNAs Arrc07, Arrc14, and Arrc20, respectively. The possible target of Arrc17, the mRNA for the gene *pbpB*, is involved in the resistance to penicillin. Two Arrc14 and one Arrc07 targets are related to cell adherence and biofilm formation; the first one may control the expression of tight adherence proteins coded by the genes *tadE* and *tadD*, and the latter may regulate the expression of the biofilm synthesis protein PgaA. Also, cell surface components involved with either adherence or protection are the possible targets of Arrc02. It may control the product of the locus APL_1273, which is a fimbrial biogenesis protein. Other sRNA candidates may control the expression of mRNA targets involved with the bacterial resistance to stressful conditions. The sRNA Arrc05 likely binds three of these mRNAs, transcribed from the genes *uspA*, *ostA*, and *recJ*. These genes encode a universal stress protein, an organic solvent tolerance protein, and an exonuclease involved with DNA damage repair, respectively. The genes *rdgC* and *radA* (likely targets of Arrc02 and Arrc11, respectively) also encode proteins that participate in processes of repair and recombination. The expression of four genes coding for heat shock proteins and/or chaperones may be controlled by Arrc07 (gene *grpE*), Arrc11 (gene *djlA*), and Arrc14 (genes *htpG* and *torD*). Finally, some targets predicted for GcvB (Arrc01), highly involved in amino acid transport and metabolism, have already been described for this sRNA (Sharma et al. 2011), such as the genes *lrp*, *ilvC*, *ilvE*, and *serA*, all linked to amino acid biosynthesis.

### Distribution of the sRNAs among *Pasteurellaceae*

Overall, 51 complete genomes available in Genbank from 15 different species of the *Pasteurellaceae* family were searched for similar RNAs sequences found in *A. pleuropneumoniae*, adopting a cutoff of 60% of identity and coverage. Among them, only *Mannheimia succinoproducens* and *Actinobacillus succinogenes* are not usually pathogenic. The distribution of the sequences of the regulatory RNA candidates described in this work ranges from 100% (23 out of 23, for all *A. pleuropneumoniae* serotype references and all the Brazilian clinical isolates) to 13% (three out of 23, for *A. actinomycetemcomitans*, *Aggregatibacter aphrophilus*, *Haemophilus parainfluenzae*, and *Pasteurella multocida*). The results are shown in Supplemental Table S3. The sequence conservation and taxonomical dispersion among the 17 additional—apart from L20—*A. pleuropneumoniae* strains (including 11 serotype reference strains and six Brazilian clinical isolates) and another 14 *Pasteurellaceae* species (comprising 33 complete genomes) are also depicted in Table 1.

None of the *A. pleuropneumoniae* putative sRNAs are exclusive to the species, as all the sequences were found (100% of distribution) in the genome of *A. suis* 130Z, which is also a pig pathogen and closest relative to *A. pleuropneumoniae*, as recently shown by phylogenomics (Naushad et al. 2015). The species of the genus *Mannheimia* are the next ones sharing the highest number of sRNA sequences with *A. pleuropneumoniae*. The strains of *Mannheimia varigena*, a bovine respiratory pathogen, present an average of 52% (12/23) of the sRNAs in common with *A. pleuropneumoniae*, while *Mannheimia haemolytica*, also a bovine respiratory pathogen, presents an average of 48% (11/23). Supplemental Figure S2 shows the sequence alignment for the novel *trans-*acting sRNAs described in this work.

Only three regulatory RNA candidates are well conserved among all the genomes analyzed: The RtT (Arrc21), the RNAseP (Arrc15), and the tmRNA (Arrc23) are present in all the *Pasteurellaceae* genomes available in at least 80% of the species. Additionally, sequences similar to GcvB are present next to the *gcvA* gene (or its homolog) identified in most of the *Pasteurellaceae* genomes analyzed (Supplemental Fig. S2).

## DISCUSSION

RNA molecules play a great variety of regulatory roles in all life domains, and increasing evidence shows they are implicated in virtually every aspect of cell metabolism (Waters and Storz 2009). This is especially important for bacterial pathogens, whose lifestyles require tight control of virulence gene expression and general stress responses (Papenfort and Vogel 2010; Bilusic et al. 2014). Several studies have revealed the existence of sRNAs particularly involved in pathogenicity. As a result, an ever-growing library of virulence-related regulatory RNAs is being established in this relatively young field of life science. As recent specific examples, there are: the sRNA RyhB, which participates in the regulation of the production of siderophores in *E. coli* (Porcheron and Dozois 2015); the *Staphylococcus aureus* sRNA teg49, whose lack hampers biofilm formation (Kim et al. 2014), the sRNA NrsZ, which modulates *Pseudomonas aeruginosa* motility (Wenner et al. 2014); and four novel sRNAs in *Enterococcus faecalis*, whose deletions affected bacterial virulence and stress tolerance when compared to the wild-type strains (Michaux et al. 2014a). CRISPR elements are usually considered as the bacterial innate immune system to face mainly bacteriophage and conjugative plasmid invasions (Koonin and Wolf 2015), and increasing evidence shows their differential expression during responses to stressful changes in the environment and during infection (Louwen et al. 2014).

For the *Pasteurellaceae* family, to our knowledge, there have been few studies investigating sRNAs. Although the relevance of sRNAs in *A. pleuropneumoniae* pathogenesis is suggested by previous work with mutants for the sRNA molecular chaperone Hfq (Subashchandrabose et al. 2013; Pereira et al. 2015b), little information exists on abundance and possible roles.

The discovery of novel regulatory RNAs has largely relied on RNA-seq experiments, which may or may not be preceded by the coimmunoprecipitation of RNAs with the Hfq chaperone (Li et al. 2013; Bilusic et al. 2014; Gomez-Lozano et al. 2014a). Bioinformatics is also an important and less expensive tool to complement or replace the searches for regulatory RNAs in microorganisms and has been used with success (Tesorero et al. 2013), including the work with the *Pasteurellaceae* human pathogen *A. actinomycetemcomitans* (Amarasinghe et al. 2012). Although the computational prediction of sRNA candidates greatly diminishes the costs and time of initial experiments, the difficulties in handling algorithms generally limits their use by most biologists. To circumvent this predicament, in this work, we propose a strategy using up-to-date programs, all hosted in free online and easy-to-use platforms, for the discovery of regulatory RNAs, using *A. pleuropneumoniae* as a model. We chose the serovar 5 strain L20 as the basis for our study because it is considered to be highly virulent, and it was the first strain for which a complete closed genome was available (Foote et al. 2008).

As reviewed previously (Sridhar and Gunasekaran 2013), many algorithms following distinct parameters for the discovery of small regulatory RNAs have been created. Since single algorithm runs can result in a very high and unrealistic number of RNA candidates to logistically screen for, as was found in this study (see below), we used four of the available algorithms, each one with a different approach, and then compared the results in order to select candidates indicated by at least two of the methods used. A similar strategy was successfully adopted and led to the discovery of seven novel sRNAs in *Streptococcus pyogenes* (Tesorero et al. 2013).

The algorithms RNAz, INFERNAL, and BLASTn were all used through the platform RNAspace (Cros et al. 2011), and SIPHT was used through its own platform (Livny 2012). RNAz is a method of comparative genomics that searches for conserved genome fragments which present sRNA motifs, while evaluating the thermodynamic stability of their secondary structure (Gruber et al. 2010). Also a comparative model, INFERNAL takes the genome sequence used as the input to build consensus RNA secondary structure profiles, called covariance models, and uses them to search nucleic acid sequence databases for homologous RNAs (Nawrocki and Eddy 2013). The simple BLASTn approach was used for searching sRNAs that have already been described and deposited in Rfam, the largest database for noncoding RNAs (Nawrocki et al. 2015). And finally, SIPHT identifies sRNA candidates by searching for conserved intergenic regions upstream of predicted intrinsic Rho-independent transcription terminators (Livny 2012). For our search with SIPHT, we used the same moderately stringent parameters as described by Livny et al. (2008), as these specific values were the ones that generated the lowest number of false sRNA candidates, taking into consideration the analyses of genomes of microorganisms whose sRNAs content had been previously studied. Because of that, SIPHT predicted about three times fewer candidates than the other three algorithms.

If not analyzed in combination, the four algorithms would have predicted 512 different RNA candidates (195 predicted exclusively by RNAz, 165 by INFERNAL, 32 by SIPHT, and 97 by BLAST, plus 23 predicted by two or more programs), a number very far from the expected for a microorganism with a genome of ∼2.2 Mb, such as *A. pleuropneumoniae*. As a comparison, in *E. coli*, whose genome is about twice as big as that of *A. pleuropneumoniae*’s genome, around 80 sRNAs have already been described (Modi et al. 2011). We would therefore expect roughly half the regulatory RNAs in our model microorganism.

Here, we predicted 23 regulatory RNA candidates and observed the expression of 17 of them in aerobic and anaerobic conditions, mostly by RT-PCR and abundance by Northern blotting. There was comparatively little overlap of the predicted sRNAs detected by each program reflecting the analytical basis underlying the individual algorithms. For example, RNAz, in contrast to SIPHT and INFERNAL, makes predictions across the whole genome and is not limited to intergenic regions. RNAz predictions, in addition, are based on a comparative analysis between limited numbers of genomes determined by the user and thus, depend on the presence of regulatory motifs in the genome of interest and their conservation in the genomes used for comparison. BLAST on Rfam relies on previously described sRNAs, which are underrepresented for the *Pasteurellaceae* family, and also selects noncoding RNAs, such as tRNAs, snoRNAs, and rRNAs, that were not the prime focus of our study. RNAz made the largest number of predictions amongst the four programs including 20 out of 23 sRNAs in intergenic regions. Arrc 19 (his riboswitch) and 22 (Mo riboswitch) are missing because of sequence variation in the three *A. pleuropneumoniae* genomes used for analysis. Arrc23 is a tmRNA which, despite being conserved, does not meet the criteria for the RNAz search, since it is structurally similar to a tRNA, with features of an mRNA.

We used aerobiosis as it is the typical atmosphere used in experiments involving *A. pleuropneumoniae* and anaerobiosis as, based on mutant (Baltes et al. 2005; Jacobsen et al. 2005; Buettner et al. 2009) or transcriptome (Deslandes et al. 2010; Klitgaard et al. 2012) studies, it is representative of the growth conditions found in necrotic lungs of pigs. As shown in Figure 4 and Table 1, some *trans*-acting sRNA candidates, like Arrc05 and Arrc08, are located between genes within operons, which could allow them to be mistaken for subproducts of polycistronic mRNA maturation. However, features of their sequences and vicinity, like promoters and transcription terminators, indicate they are indeed sRNAs. Their functional characterization, which is underway, is a tougher task to perform, since producing mutants for these genes could interfere with the expression of the operon where they are located.

Only 9/23 RNAs (39%) were also identified by RNA-seq, which could be explained by the low number of reads obtained and by sequencing errors inherent of the IonTorrent platform (Mardis 2013). It is possible that the use of a different new generation sequencing platform allowing a greater number of reads and/or accuracy would have identified the remaining 14 sRNAs. However, our results suggest that prediction of sRNA by software can be informative. For *E. coli*, the minimum of 2 million reads from an IlluminaHiSeq sequencer was necessary to cover (at least one time) 96% of each expectedly expressed ORF (Haas et al. 2012). Therefore, it is still possible that some of the six undetected RNAs exist but are not expressed, or are expressed in low levels, in the conditions tested. Nevertheless, the strategy proposed herein had an accuracy of almost 74%, slightly raising the 72% accuracy of the suggested SIPHT parameters alone (Livny et al. 2008), and more than twofold higher than the 31% accuracy of a similar approach used for *S. pyogenes*, in which 14 out of 45 RNA candidates predicted by the combination of three different algorithms had their expression confirmed (Tesorero et al. 2013). Since this is one of the first efforts to discover regulatory RNAs in *A. pleuropneumoniae*, the transcripts observed in this work greatly contribute to the understanding of novel genes in this microorganism's genome and in those of the family. Even more remarkable is the fact that many of these RNAs have no homologues in the Rfam database, therefore raising the global repertoire of regulatory noncoding RNA. All four algorithms will undoubtedly benefit from an increase in the number of confirmed sRNAs documented in the *Pasteurellaceae*. This is the first description of CRISPR-associated spacer sequences for *A. pleuropneumoniae*, although their features will be better studied in a future work. It remains to be elucidated if the system is still active in this bacterium, because despite having several genes for the typical Cas proteins, some essential expected proteins, such as the spacer-acquisition protein Cas2 (van der Oost et al. 2014), are absent.

As expected for a bacterial pathogen, the overall 23 sRNAs include regulatory molecules with important implications for bacterial cell maintenance and pathogenesis. Among them, one of the most widespread and abundant (approximately 10,000 copies per cells in stationary phase) is the 6S RNA, a global regulator sRNA that reduces the expression of several σ-70 dependent promoters, favoring the interaction of RNA polymerase with alternate sigma factors, such as RpoS in *Bacillus subtilis* (Cavanagh and Wassarman 2014), and has been implicated in the down-regulation of the expression of key pathways in response to changing stressful conditions and growth adaptation (Cavanagh et al. 2010; Cavanagh and Wassarman 2013). GcvB is also one of the most highly conserved Hfq-associated sRNAs in Gram-negative bacteria and was previously reported to regulate many genes involved in the transport and biosynthesis of oligopeptides and amino acids, such as the branched-chain amino acid (BCAA) transport system (Sharma et al. 2011; Stauffer and Stauffer 2013). The BCAA biosynthesis and transport system is well studied in *A. pleuropneumoniae*. The presence of these amino acids is required for the survival of the bacterium and their lack is responsible for the expression of both genes for their own biosynthesis, and virulence-related genes, as demonstrated in pigs (Wagner and Mulks 2006; Subashchandrabose et al. 2009). GcvB is also known to regulate the PhoQ–PhoP two-component system, which is involved in magnesium homeostasis, pathogenicity, cell envelope composition, and acid resistance in several bacterial species (Coornaert et al. 2013). Also involved in stress response, the sRNAs of the RtT family were first discovered as oligonucleotides released from the primary transcript of the tyrT and many other tRNA operons in *E. coli* during the tRNA processing. These sRNAs present modulatory effects on the stringent response and are overproduced during cell contact with antibiotics (Bosl and Kersten 1991; Kohanski et al. 2007). The *cis*-acting RNA FMN has also been implicated in oxidative stress resistance in *Deinococcus radiodurans* (Yang et al. 2014).

A total of 12 *trans*-acting sRNAs were described, and eight of them are novel regulators whose roles in the bacterial cell are unknown. As it has been proposed before (Gruber and Sperandio 2015; Kim et al. 2015; Peng et al. 2015), computational analysis of the sRNAs’ targets is an excellent starting point toward understanding their physiological roles in the cell. Here, we used TargetRNA2 as a target predictor for being the algorithm with the best correlation of targets predicted and actually confirmed, among the programs widely used for this purpose (Kery et al. 2014). Because many of the mRNAs predicted are potential targets of more than one sRNA, these regulators may share some of their targets, placing them in a characteristic entangled network of gene regulation (Modi et al. 2011). Our predictions are strongly corroborated by the fact that several of the targets predicted for the GcvB (Arrc01) sRNA had been shown for other microorganisms and are consistent with its role (Sharma et al. 2011). Most sRNAs described in bacteria to date are negative regulators of gene expression but a small group of sRNAs (DsrA, GlmZ, RNAIII, RprA, RyhB, and Qrr) has been shown to act directly as translational activators (Fröhlich and Vogel 2009, Soper et al. 2010). We envisage that the Arccs discovered in our study are negative regulators, although further experiments, e.g., comparative RNA-seq with wild-type and mutants for each Arrc are required to clarify the relationship of each sRNA and their cognate mRNAs.

Most sRNAs have as targets mRNAs directly implicated in either virulence or stress resistance. Although this was partially expected given the aforementioned phenotypes of reduced biofilm-forming capacity, sensitivity to oxidative stress (Subashchandrabose et al. 2013), and reduced virulence in an alternative infection model (Pereira et al. 2015b) displayed by *A. pleuropneumoniae* mutants for the *hfq* gene, new candidates for virulence determinants were defined by the present regulatory network. Two categories of potential targets that are part of extremely important systems for the establishment of *A. pleuropneumoniae* as a pig pathogen, and thus strong mutant candidates for live attenuated vaccines, are the iron-acquisition systems and Apx toxins. While the ability of *A. pleuropneumoniae* to overcome iron-restriction is essential to pathogen permanency in the host, the pore-forming and cytolytic Apx toxins are directly related to the pathology of porcine pleuropneumonia (Jacques 2004; Frey 2011). sRNAs are enriched in intergenic regions, which are longer and more conserved than the average intergenic regions in bacterial genomes (Tsai et al. 2015). For that reason, we performed a homology search of the sRNAs sequences identified for *A. pleuropneumoniae*, in the available complete genomes of other bacteria of the *Pasteurellaceae* family. Contrary to expectations, the novel sRNAs found in *A. pleuropneumoniae* are not widespread in *Pasteurellaceae*. The most widely distributed sequences in the family are the ones of housekeeping regulatory RNAs, whose functions are essential to the bacterium, such as the RNAseP (Arrc15) and tmRNA (Arrc23). *Actinobacillus suis* is the only pathogen included in this analysis that shares all the candidates’ sequences with *A. pleuropneumoniae*, which can be explained by the evolutionary closeness of these species as recently reinforced by phylogenomics (Naushad et al. 2015). However, unlike *A. pleuropneumoniae*, *A. suis* is not a primary pathogen but an opportunistic one that can also cause disease in pigs of all ages (Christensen and Bisgaard 2004; MacInnes et al. 2012). Besides their putative sRNAs, both pathogens have many virulence factors in common, which makes *A. suis* also capable of causing a hemorrhagic pleuropneumonia, though it most often causes septicemia and diseases such as arthritis and meningitis that are sequelae to septicemia (Ojha et al. 2010).

The other *Pasteurellaceae* with substantial numbers of RNA candidate sequences in their genome in common with *A. pleuropneumoniae* are *M. haemolytica*, *M. varigena*, and *H. parasuis*. The species of the genus *Mannheimia* are relatively close to *A. pleuropneumoniae* as seen by the family's phylogenomics, though the same is not true for *H. parasuis*. Both *M. haemolytica* and *M. varigena* are occasional respiratory pathogens of cattle and pigs and a transcriptome study with *M. haemolytica* had already shown one putative sRNA in common with *A. pleuropneumoniae* (Reddy et al. 2012; Harhay et al. 2014). The similarities between *A. pleuropneumoniae* and *H. parasuis* may be explained by the fact that they share the same host. *H. parasuis* is one of the earliest and most prevalent colonizers of piglets in the farrowing house, a commensal of the respiratory tract, a common isolate from nasal secretions in pigs, and the cause of Glässers disease (Xu et al. 2011). It is expected that some of the sRNAs identified in this study might be important for the establishment of these bacteria within the porcine host and even for causing disease.

In conclusion, this work is one of the few to base the initial search of bacterial regulatory RNAs exclusively on bioinformatics, an inexpensive and faster alternative to the most commonly used methods for sRNAs discovery. The success of the strategy proposed herein is justified mainly by the use of up-to-date algorithms with different approaches easy to manipulate, showing that our strategy will be useful for the elucidation of novel regulatory RNAs in microbial genomes. Our findings are also a great step forward in the understanding of the coding potential of *A. pleuropneumoniae* and of the *Pasteurellaceae* family in general. Because many of the RNAs discovered are potentially involved in virulence, it is possible that mutants for those sRNAs can be effectively used as attenuated vaccines, providing new horizon for further studies.

## MATERIALS AND METHODS

An overview of the computational and experimental strategies used to identify and characterize novel sRNAs in *A. pleuropneumoniae* are summarized in Figure 1 and explained with details below.

### Bacterial strains and RNA extraction

The experiments were conducted with the *A*. *pleuropneumoniae* serotype 5 reference strain L20. The strain was grown in Brain Heart Infusion (BHI) supplemented with nicotinamide adenine dinucleotide (NAD—10 µg ml^−1^) at 37°C until early stationary phase (8 h) under aerobic (5% CO_2_) and anaerobic conditions (anaerobic jar with Oxoid Anaerogen Sachet, Thermo Scientific). Total RNA extraction was performed by cell disruption using the Lysing Matrix B tubes (MP Biomedicals), followed by the procedures of the miRNeasy Mini Kit (QIAGEN), according to the manufacturer's instructions. After extraction, the concentration and purity of the RNA was determined by Nanodrop and 2100 Bioanalyzer (Agilent Technologies). The resulting total RNA (purity 1.8–1.9, A260/A280 ratio) was treated with one unit of RQ1 DNAse (Promega) per µg of nucleic acid, and incubated for 60 min at 37°C.

### In silico identification of sRNAs

The computational searches for discovery of sRNAs were performed with the genome of *A. pleuropneumoniae* strain L20 (Genbank access NC_009053). Four algorithms with distinct approaches were used to increase prediction accuracy: RNAz (Gruber et al. 2010), INFERNAL (Nawrocki and Eddy 2013), SIPHT (Livny 2012), and BLASTn on Rfam (Nawrocki et al. 2015). The predictions with BLASTn, RNAz, and INFERNAL were performed through the RNAspace platform (Cros et al. 2011), available at www.rnaspace.org/. The BLASTn approach consisted in performing a homology search against all the regulatory RNAs sequences available from the Rfam 10.0 database. The RNAz run was made with the default values, i.e., probability cutoff: 0.7, slice alignments longer than: 300, window size: 200, step size: 50. Genome-wide sequence alignment of *A. pleuropneumonie* L20 was made with the annotated genomes of *A. pleuropneumoniae* JL03 and AP76, *Haemophilus ducreyi* 35000HP, and *H. influenzae* PittEE (Genbank accesses NC_010278, NC_010939, NC_002940, and CP000671, respectively) through BLAST, filtering low complexity regions in both strands of query sequence and adopting an *E*-value threshold of 0.001, which are the default values in RNAspace. CG-seq was used for sequence aggregation, also using the RNAspace default parameters, score lambda parameter: 1, minimal and maximal length of a conserved region: 30 and 500, minimum and maximum identity threshold: 60 and 100. For the prediction with INFERNAL, the query genome sequence was used to build a covariance model (CM), then used to search homologous RNAs sequences on the Rfam 10.0 database. For the SIPHT run, whose platform is available at http://newbio.cs.wisc.edu/sRNA/, the moderate stringency parameters suggested by the author were used, which are as follows; maximum *E*-value: 1 × 10^−15^, minimum TransTerm confidence value: 87%, maximum RNAMotif score: −9, FindTerm scores: −10, and minimum and maximum lengths of predicted loci: 50 and 500 (Livny et al. 2008). Finally, the resulting RNA lists of each algorithm were compared against each other with BLASTn, and the sequences that were predicted by at least two of the four methods applied were considered as sRNA candidates. For the identification of CRISPRs in the genome of the *A. pleuropneumoniae* L20, the standard definitions of the software CRISPRFinder (Grissa et al. 2007) were used.

### Taxonomical dispersion of the *A. pleuropneumoniae*’s sRNAs in the *Pasteurellaceae* family

The sequences of the final sRNA candidates were also searched by BLASTn in the other 11 serotype reference genomes available of *A. pleuropneumoniae* and in the genomes of six Brazilian clinical isolates (Pereira et al. 2015a) to investigate their distribution among the species. In addition, 33 genomes of 14 other *Pasteurellaceae* species, whose complete genomes are available from Genbank, were also searched (Supplemental Table S4).

### Reverse transcription polymerase chain reaction (RT-PCR)

To identify the coding strand of each predicted regulatory RNA candidate, RT-PCR was performed. For cDNA synthesis, the ImProm-II Reverse Transcription System (Promega) was used, according to the manufacturer's instructions, using either the forward or the reverse primer designed for each sRNA candidate separately (Supplemental Table S1). The cDNA reaction (20 µL) was further used in a PCR reaction using the primer pair for each sRNA. The PCR reaction was performed with 1 U of GoTaq DNA polymerase (Promega) in a final volume of 50 µL of enzyme buffer containing 1.5 mM MgCl_2_, 0.2 mM of each dNTP, and 0.2 µM of each primer in a thermal cycler Mastercycler pro (Eppendorf). The samples were initially denatured at 94°C for 2 min, followed by 35 reaction cycles (94°C for 1 min, 55°C for 1 min, and 72°C for 30 sec) and a final extension step at 72°C for 5 min. The amplicons generated were analyzed after electrophoresis in a 2.0% agarose gel. As a positive amplification control, 50 ng of *A. pleuropneumoniae* L20 genomic DNA was used as template and as negative control, a reaction with DNA-free total RNA that was not subjected to the reaction of cDNA synthesis was used.

### Northern blotting

Total RNA (10 µg) was run on a 10% TBE-urea gel and transferred to a Brightstar Plus nylon membrane (Applied Biosystems). Hybridization was conducted with the DIG High Prime DNA Labeling and Detection Starter kit II (Roche), according to the manufacturer's instructions. The oligonucleotide pairs used for the RT-PCR reaction (Supplemental Table S1) were also used to construct 126 ± 27 bp digoxigenin-marked probes designed for the inner parts of the sRNA candidates’ sequences with the PCR DIG probe synthesis kit (Roche). As hybridization controls we used dot blots with total DNA and all membranes used were hybridized with probes for the rRNA 5S, using the primer pair APP5SF/APP5SR (Supplemental Table S1).

### RNA sequencing, reads mapping and transcriptome assembly

Total RNA was extracted from *A. pleuropneumoniae* MIDG2331, a clinical isolate from the UK, as described above and was treated with the MICROBExpress Kit (Life Technologies) for ribosomal RNA removal. The cDNA library construction for both aerobic and anaerobic conditions was carried out using the Ion Total RNA-Seq Kit v2 (Life Technologies) according to manufacturer's protocols. Samples were loaded onto a 318 chip and sequenced on Ion torrent-PGM (Life technologies) using default parameters (single-end, forward sequencing). The sequenced reads were mapped to the *A. pleuropneumoniae* L20 reference strain genome using Burrows-Wheeler Aligner (BWA-MEM algorithm, default parameters) version 0.7.10 (Li and Durbin 2009). The resulting bam files were uploaded in NCBI-Short Read Archive (SRA) under the experiment access SRX810211. Transcriptome assembly was made with Cufflinks version 2.2.1 (Trapnell et al. 2012).

### Investigation of putative mRNA targets

The potential mRNAs targets of the putative novel *trans*-acting RNA (sRNA) candidates identified were searched for every annotated gene on the *A. pleuropneumoniae* L20 genome. Searches were performed with the software TargetRNA2 (Kery et al. 2014), considering the conservation (compared to every sequenced replicon available in GenBank) and accessibility of each sRNA given as input, structural accessibility of the mRNA and potential interactions preceded by a hybridization seed around the translation start site, from 80 nt upstream to 20 nt downstream from it. Only target interactions with a *P*-value less than or equal to 0.05 were reported. Potential mRNA targets shared by the sRNAs were identified and used as the basis for the design of a regulatory network comprising each sRNA and their putative targets.

## SUPPLEMENTAL MATERIAL

Supplemental material is available for this article.

## Supplementary Material

Supplemental Material
