# Supplementary material for: A computational strategy for the search of regulatory small RNAs in Actinobacillus pleuropneumoniae
Source: RNA. 2016 Sep;22(9):1373–85. doi: 10.1261/rna.055129.115 (PMC4986893; doi:10.1261/rna.055129.115)
Supplement: Supplemental Material [file supp_22_9_1373__index.html]

A computational strategy for the search of regulatory small RNAs in Actinobacillus pleuropneumoniae — A computational strategy for the search of regulatory small RNAs in Actinobacillus pleuropneumoniae — Supplemental Material 

# A computational strategy for the search of regulatory small RNAs in *Actinobacillus pleuropneumoniae*

## Supplemental Material

- Supplemental\_Material.docx
