# Supplementary material for: A computational strategy for the search of regulatory small RNAs in Actinobacillus pleuropneumoniae
Source: RNA. 2016 Sep;22(9):1373–85. doi: 10.1261/rna.055129.115 (PMC4986893; doi:10.1261/rna.055129.115)
Supplement: Supplemental Material [file supp_055129.115_Supplemental_Material.docx]

**SUPPLEMENTAL MATERIAL**

| **Table S1** Oligonucleotides designed for *Actinobacillus pleuropneumoniae* regulatory RNA candidates used in this work. | | | |
| --- | --- | --- | --- |
| **sRNA target** | **Primer name** | **5’🡪3’ sequence** | **Amplicon (bp)** |
| Arrc01 | ARRC01F | TGTTGTGTTTGCATATTGGTCTAGG | 122 |
|  | ARRC01R | TGGACGGTTATAAACCAAAAAGGT |  |
| Arrc02 | ARRC02F | TGCTGATTTCAAGGTAAAAGCG | 130 |
|  | ARRC02R | GGCTTAAAAGACGAGGGCGA |  |
| Arrc03 | ARRC03F | AGGAAAGGCGTATTTGCCGA | 119 |
|  | ARRC03R | GAACCGACCCTAGCAGTAGC |  |
| Arrc04 | ARRC04F | CGCAAAAAGTGCTTGCATTGG | 140 |
|  | ARRC04R | GCCTTAAAACTGGTTGCGGG |  |
| Arrc05 | ARRC05F | CGGTGTGTAAGCGGTCTGAT | 103 |
|  | ARRC05R | GGATACCGAGCTTGTATGCCT |  |
| Arrc06 | ARRC06F | ATGGGGCGTTATTGGTTCCT | 105 |
|  | ARRC06R | CGTTACCAGCAACCCTCGG |  |
| Arrc07 | ARRC07F | AGGTAGCTGGAGAAGAGCGA | 182 |
|  | ARRC07R | TTCTCCCCTGTCCTTTTGCC |  |
| Arrc08 | ARRC08F | AGAGCAAGCTGATGGTGCTT | 160 |
|  | ARRC08R | CGCTTGCATCGCAAGTAGC |  |
| Arrc09 | ARRC09F | AACCGCTTGTCATGAACTCC | 121 |
|  | ARRC09R | AGAAAAGAGGGGTTAGGGGA |  |
| Arrc10 | ARRC10F | CCGTTACTTGTGGTGGTCCT | 153 |
|  | ARRC10R | TGCGTTACTCGTTACGTCCC |  |
| Arrc11 | ARRC11F | TGTCCAATAAATAGGCTTCCCA | 126 |
|  | ARRC11R | AACTATCCAAATAAAAAGTACGGCT |  |
| Arrc12 | ARRC12F | CTACAGGCACATTTCGCAGC | 115 |
|  | ARRC12R | CGCTTATCGCTAACCGTCTT |  |
| Arrc13 | ARRC13F | ATAATCTTCAGGGCAGGGTGA | 100 |
|  | ARRC13R | ACTGTCGGTACCGGAATCTC |  |
| Arrc14 | ARRC14F | ACGACTATCTCTTCGACTGCT | 103 |
|  | ARRC14R | GCATCAATGTGCGGGCAAAG |  |
| Arrc15 | ARRC15F | AGGAACTCAATGGATGGCCC | 106 |
|  | ARRC15R | TCGATAAGCCGAGTTCTGTCG |  |
| Arrc16 | ARRC16F | ACGGGATACATTGGAATTGATAAGG | 100 |
|  | ARRC16R | TAGGTAATCACTCCAACTCTTACGC |  |
| Arrc17 | ARRC17F | TTCTTTCTTGCAAAGAACCCGC | 100 |
|  | ARRC17R | ATGCTGATCTTGAAAAGCCCG |  |
| Arrc18 | ARRC18F | ACGATGAGTCGCAAATTCCC | 124 |
|  | ARRC18R | AAAGAGAAACTCCGCACAACA |  |
| Arrc19 | ARRC19F | TTCGGGCATTTGAGTGTGGT | 101 |
|  | ARRC19R | AGTTGCCTTCCGAGAGGTTG |  |
| Arrc20 | ARRC20F | GCATTTGACGCTAAAACGGT | 128 |
|  | ARRC20R | AATTAGTGGCTCCTCCTGCG |  |
| Arrc21 | ARRC21F | GACCCTTTAGAAGGCGTTGC | 115 |
|  | ARRC21R | CGCAACGTTAAGGGTCGTTAG |  |
| Arrc22 | ARRC22F | GACTCCGAGCTTGTGAACCT | 176 |
|  | ARRC22R | TGGATTGCATTGGACACCTT |  |
| Arrc23 | ARRC23F | TGGATTCGACGGGATTAGCG | 179 |
|  | ARRC23R | TGGGTGACTTATCGTTGCCC |  |
| rRNA 5S | APP5SF | GCGATGCCCTACTCTCACAT | 100 |
|  | APP5SR | GAGTGCTGTGGCTCTACCTG |  |

| **Table S2** Potential mRNA targets of the putative *Actinobacillus pleuropneumoniae* novel *trans*-acting RNAs described in this work. Putative binding positions in target mRNA 5’ UTR were determined using TargetRNA2 ([Kery et al. 2014](#_ENREF_34)). | | | | | | | |
| --- | --- | --- | --- | --- | --- | --- | --- |
| **RNA ID** | **Rank** | **Targetgene** | **Locus** | **Energy (kcal/mol)** | ***p*-value** | **Putative binding position in target mRNA*** | **Description** |
| **Arrc01** | 1 | *arcD* | APL_1082 | -18.62 | 0.000 | -29 to -14 | arginine/ornithine antiporter |
|  | 2 | *ilvC* | APL_1853 | -18.15 | 0.000 | -62 to -44 | ketol-acid reductoisomerase |
|  | 3 | *ilvI* | APL_0727 | -15.74 | 0.000 | -41 to -26 | acetolactate synthase 3 catalytic subunit |
|  | 4 | *dapE* | APL_1873 | -14.6 | 0.001 | +3 to 19 | succinyl-diaminopimelate desuccinylase |
|  | 5 | *thrC* | APL_1499 | -13.03 | 0.004 | +5 to 20 | threonine synthase |
|  | 6 | *fabG* | APL_1992 | -12.73 | 0.005 | -67 to -53 | 3-ketoacyl-ACP reductase |
|  | 7 | *ilvD* | APL_0097 | -12.55 | 0.005 | -32 to -20 | dihydroxy-acid dehydratase |
|  | 8 | *menA* | APL_1461 | -12.1 | 0.007 | -19 to -5 | 1,4-dihydroxy-2-naphthoate octaprenyltransferase |
|  | 9 | ***hisD*** | APL_2020 | -11.53 | 0.010 | -9 to 9 | histidinol dehydrogenase |
|  | 10 | *serC* | APL_0702 | -11.42 | 0.011 | -10 to +6 | phosphoserine aminotransferase |
|  | 11 | *ftsK* | APL_0618 | -11.34 | 0.011 | -48 to -37 | DNA translocase FtsK |
|  | 12 | *dxs* | APL_0207 | -11.04 | 0.013 | -5 to +12 | 1-deoxy-D-xylulose-5-phosphate synthase |
|  | 13 | *plpA* | APL_0910 | -10.77 | 0.016 | -32 to -15 | outer membrane lipoprotein 1 |
|  | 14 | *ureA* | APL_1618 | -10.64 | 0.017 | -28 to -15 | urease subunit gamma |
|  | 15 | *sfsA* | APL_1737 | -10.55 | 0.018 | -8 to +7 | sugar fermentation stimulation protein A |
|  | 16 | ***hisC*** | APL_2021 | -10.32 | 0.020 | 2 to 20 | histidinol-phosphate aminotransferase |
|  | 17 | *serB* | APL_1230 | -9.95 | 0.024 | -2 to +15 | phosphoserine phosphatase |
|  | 18 | *dapA* | APL_0899 | -9.72 | 0.027 | -21 to -2 | dihydrodipicolinate synthase |
|  | 19 | ***hisI*** | APL_2028 | -9.65 | 0.028 | -9 to 12 | bifunctional phosphoribosyl-AMP cyclohydrolase/phosphoribosyl-ATP pyrophosphatase |
|  | 20 | *gcp* | APL_1120 | -9.49 | 0.030 | -10 to +9 | DNA-binding/iron metalloprotein/AP endonuclease |
|  | 21 | *hisG* | APL_2019 | -9.21 | 0.034 | -25 to -10 | ATP phosphoribosyltransferase |
|  | 22 | *leuC* | APL_0139 | -9.14 | 0.035 | -46 to -26 | isopropylmalate isomerase large subunit |
|  | 23 | *gntR* | APL_1667 | -9.02 | 0.037 | -3 to +15 | HTH-type transcriptional regulator |
|  | 24 | *secE* | APL_1716 | -8.87 | 0.039 | -79 to -64 | preprotein translocase subunit SecE |
|  | 25 | *lrp* | APL_0617 | -8.66 | 0.043 | -76 to -63 | leucine-responsive transcriptional regulator |
|  | 26 | *ilvE* | APL_0072 | -8.5 | 0.046 | -12 to +8 | branched-chain amino acid aminotransferase |
|  | 27 | *uxaC* | APL_1020 | -8.49 | 0.046 | -43 to -30 | uronate isomerase |
|  | 28 | *serA* | APL_1452 | -8.4 | 0.048 | -24 to -10 | D-3-phosphoglycerate dehydrogenase |
|  | 29 | *tehB* | APL_1350 | -8.35 | 0.049 | -8 to +10 | tellurite resistance protein TehB |
|  | 30 | ***trpD*** | APL_1165 | -8.35 | 0.049 | -69 to -54 | anthranilate phosphoribosyltransferase |
| **Arrc02** | 1 | *ilvH* | APL_0728 | -15.37 | 0.000 | -15 to -2 | acetolactate synthase 3 regulatory subunit |
|  | 2 | *ptnD* | APL_1393 | -14.49 | 0.001 | -18 to -3 | PTS system mannose-specific transporter subunit IID |
|  | 3 | *plsB* | APL_1107 | -13.65 | 0.002 | -80 to -64 | glycerol-3-phosphate acyltransferase |
|  | 4 | *-* | APL_1273 | -13.22 | 0.003 | +5 to +19 | fimbrial biogenesis and twitching motility protein PilF-like protein |
|  | 5 | ***murI*** | APL_1841 | -12.8 | 0.004 | -62 to -48 | glutamate racemase |
|  | 6 | *rumB* | APL_1112 | -12.45 | 0.006 | +6 to +19 | 23S rRNA methyluridine methyltransferase |
|  | 7 | *gmhA* | APL_1364 | -12.21 | 0.007 | -76 to -67 | phosphoheptose isomerase |
|  | 8 | ***hisD*** | APL_2020 | -11.19 | 0.012 | -65 to -63 | histidinol dehydrogenase |
|  | 9 | ***rpmE*** | APL_0982 | -10.99 | 0.014 | -51 to -39 | 50S ribosomal protein L31 |
|  | 10 | *trpA* | APL_0470 | -10.88 | 0.015 | -80 to -92 | tryptophan synthase subunit alpha |
|  | 11 | *kpsF* | APL_1576 | -10.73 | 0.016 | -15 to -4 | arabinose-5-phosphate isomerase |
|  | 12 | *glpC* | APL_0381 | -10.6 | 0.017 |  | sn-glycerol-3-phosphate dehydrogenase subunit C |
|  | 13 | *thiQ* | APL_1320 | -10.47 | 0.018 | +5 to +19 | thiamine transport ATP-binding protein ThiQ |
|  | 14 | *crr* | APL_1324 | -10.41 | 0.019 | -15 to +1 | PTS system glucose-specific transporter |
|  | 15 | *eno* | APL_1113 | -10.39 | 0.019 | +1 to +16 | phosphopyruvate hydratase |
|  | 16 | *gnd* | APL_1305 | -10.32 | 0.020 | -15 to -1 | 6-phosphogluconate dehydrogenase |
|  | 17 | *rpoZ* | APL_1826 | -10.18 | 0.021 | -49 to -36 | DNA-directed RNA polymerase subunit omega |
|  | 18 | *napC* | APL_1425 | -10.12 | 0.022 | -10 to +5 | cytochrome c-type protein NapC |
|  | 19 | *rdgC* | APL_0161 | -9.87 | 0.025 | -45 to -30 | recombination associated protein |
|  | 20 | *ompP4* | APL_0389 | -9.86 | 0.025 | -11 to -1 | lipoprotein E |
|  | 21 | *recQ* | APL_1116 | -9.62 | 0.028 | -11 to +1 | ATP-dependent DNA helicase RecQ |
|  | 22 | *rnfA* | APL_0165 | -9.6 | 0.028 | -67 to -49 | Na(+)-translocating NADH-quinone reductase subunit E |
|  | 23 | *prsA* | APL_0775 | -9.39 | 0.031 | -69 to -52 | ribose-phosphate pyrophosphokinase |
|  | 24 | *pheA* | APL_1033 | -9.38 | 0.031 | -68 to -51 | P-protein |
| *Position of target mRNA interaction with sRNA given in relation to +1 translation starting point, in accordance to the transcriptome of *Actinobacillus pleuropneumoniae* reference strain L20 (Genbank access NC_009053), considering the region around the 5’UTR of each mRNA. Target genes depicted in bold are located within operons and thus the putative region of interaction between the sRNA and the mRNA is in the interior of the polycistronic RNA. | | | | | | | |
|  |  |  |  |  |  |  |  |
| **Table S2 (cont.)** Potential mRNA targets of the putative *Actinobacillus pleuropneumoniae* novel *trans*-acting RNAs described in this work. Putative binding positions in target mRNA 5’ UTR were determined using TargetRNA2 ([Kery et al. 2014](#_ENREF_34)). | | | | | | | |
| **RNA ID** | **Rank** | **Targetgene** | **Locus** | **Energy (kcal/mol)** | ***p*-value** | **Putative binding position in target mRNA*** | **Description** |
|  |  |  |  |  |  |  |  |
|  | 26 | *accB* | APL_1864 | -9.24 | 0.034 | -77 to -68 | biotin carboxyl carrier protein of acetyl-CoA carboxylase (BCCP) |
|  | 28 | *arcD* | APL_1083 | -9.11 | 0.036 | -55 to -39 | arginine/ornithine antiporter |
|  | 30 | *cpdB* | APL_0646 | -8.86 | 0.040 | -60 to -48 | bifunctional 2',3'-cyclic nucleotide 2'-phosphodiesterase/3'-nucleotidase periplasmic protein |
|  | 31 | *udp* | APL_1839 | -8.78 | 0.041 | +2 to +15 | uridine phosphorylase |
|  | 32 | *csrA* | APL_0653 | -8.44 | 0.047 | -11 to +3 | carbon storage regulator |
|  | 33 | *scrK* | APL_2034 | -8.35 | 0.049 | +4 to +17 | aminoimidazole riboside kinase |
|  | 34 | *luxS* | APL_1216 | -8.35 | 0.049 | -62 to -44 | S-ribosylhomocysteinase |
|  | 35 | *fabG* | APL_1992 | -8.34 | 0.049 | -38 to -24 | 3-ketoacyl-ACP reductase |
|  | 37 | *oapB* | APL_1404 | -8.33 | 0.049 | -17 to -6 | opacity associated protein B |
| **Arrc04** | 1 | *utp* | APL_1619 | -13.4 | 0.003 | -75 to -62 | urea transport protein ApUT |
|  | 2 | *miaA* | APL_1960 | -10.92 | 0.014 | +7 to +20 | tRNA delta(2)-isopentenylpyrophosphate transferase |
|  | 3 | *fabZ* | APL_0408 | -10.84 | 0.015 | -17 to -4 | (3R)-hydroxymyristoyl-ACP dehydratase |
|  | 4 | *valS* | APL_1502 | -10.74 | 0.016 | +5 to +14 | valyl-tRNA synthetase |
|  | 5 | *fumC* | APL_1757 | -10.65 | 0.017 | -69 to -57 | fumarate hydratase |
|  | 6 | ***malE*** | APL_1237 | -10.2 | 0.021 | -26 to -14 | maltose ABC transporter periplasmic protein |
|  | 7 | *ccp* | APL_1379 | -10.09 | 0.022 | -68 to -58 | cytochrome c peroxidase |
|  | 8 | *cysJ* | APL_1843 | -9.85 | 0.025 | -2 to +13 | sulfite reductase [NADPH] flavoprotein alpha-component (SIR-FP) |
|  | 9 | *cysB* | APL_0133 | -9.84 | 0.025 | -62 to -54 | transcriptional regulator CysB |
|  | 10 | *mioC* | APL_1563 | -9.82 | 0.026 | -13 to +2 | flavodoxin |
|  | 11 | *rimK* | APL_0484 | -9.8 | 0.026 | -76 to -62 | ribosomal protein S6 modification protein |
|  | 12 | *gnd* | APL_1305 | -9.77 | 0.026 | -23 to -8 | 6-phosphogluconate dehydrogenase |
|  | 13 | ***moaC*** | APL_0691 | -9.64 | 0.028 | +1 to +15 | molybdenum cofactor biosynthesis protein C |
|  | 14 | *crr* | APL_1324 | -9.63 | 0.028 | -11 to +2 | PTS system glucose-specific transporter |
|  | 15 | *fadI* | APL_0887 | -9.47 | 0.030 | -39 to -25 | 3-ketoacyl-CoA thiolase |
|  | 16 | *pheT* | APL_0609 | -9.25 | 0.033 | -12 to +7 | phenylalanyl-tRNA synthetase subunit beta |
|  | 17 | *kpsF* | APL_1576 | -9 | 0.037 | -14 to +6 | arabinose-5-phosphate isomerase |
|  | 18 | *nusA* | APL_0638 | -8.81 | 0.040 | +8 to +18 | transcription elongation factor NusA |
|  | 19 | *mipB* | APL_0062 | -8.8 | 0.041 | -3 to +8 | transaldolase B |
|  | 20 | *rimO* | APL_1636 | -8.78 | 0.041 | -3 to +12 | 30S ribosomal protein S12 methylthiotransferase |
|  | 21 | *tyrR* | APL_0797 | -8.67 | 0.043 | -69 to -53 | transcriptional regulatory protein TyrR |
|  | 22 | *argD* | APL_0244 | -8.67 | 0.043 | +1 to +19 | acetylornithine aminotransferase |
|  | 23 | *bioA* | APL_0942 | -8.62 | 0.044 | +1 to +13 | adenosylmethionine-8-amino-7-oxononanoate aminotransferase |
|  | 24 | *ulaD* | APL_1698 | -8.5 | 0.046 | +3 to +13 | 3-keto-L-gulonate-6-phosphate decarboxylase |
|  | 25 | *nrfB* | APL_0101 | -8.49 | 0.046 | -63 to -51 | cytochrome c nitrite reductase pentaheme subunit |
| **Arrc05** | 1 | *glmS* | APL_1631 | -17.08 | 0.000 | -78 to -63 | glucosamine--fructose-6-phosphate aminotransferase |
|  | 2 | *trkH* | APL_1590 | -15.99 | 0.000 | -54 to -35 | Trk system potassium uptake protein TrkH |
|  | 3 | *rnb* | APL_0757 | -15.07 | 0.001 | -80 to -67 | exoribonuclease II |
|  | 4 | *recJ* | APL_0459 | -13 | 0.004 | -32 to -14 | single-stranded-DNA-specific exonuclease RecJ |
|  | 5 | *arcD* | APL_1082 | -12.74 | 0.005 | -44 to -30 | arginine/ornithine antiporter |
|  | 6 | *accC* | APL_1865 | -12.53 | 0.005 | -63 to -47 | acetyl-CoA carboxylase biotin carboxylase subunit |
|  | 7 | *dut* | APL_1968 | -11.22 | 0.012 | -27 to -15 | deoxyuridine 5'-triphosphate nucleotidohydrolase |
|  | 8 | *dnaG* | APL_1474 | -10.38 | 0.019 | -32 to -17 | DNA primase |
|  | 9 | *rnpA* | APL_1939 | -10.31 | 0.020 | -80 to -66 | ribonuclease P |
|  | 10 | *xseA* | APL_0817 | -10.06 | 0.023 | -16 to -4 | exodeoxyribonuclease VII large subunit |
|  | 11 | *gpt* | APL_0255 | -10.03 | 0.023 | -19 to -3 | xanthine-guanine phosphoribosyltransferase |
|  | 12 | *sbcB* | APL_0673 | -9.99 | 0.023 | -56 to -40 | exonuclease I |
|  | 13 | *hemE* | APL_0112 | -9.87 | 0.025 | -45 to -30 | uroporphyrinogen decarboxylase |
|  |  |  |  |  |  |  |  |
| *Position of target mRNA interaction with sRNA given in relation to +1 translation starting point, in accordance to the transcriptome of *Actinobacillus pleuropneumoniae* reference strain L20 (Genbank access NC_009053), considering the region around the 5’UTR of each mRNA. Target genes depicted in bold are located within operons and thus the putative region of interaction between the sRNA and the mRNA is in the interior of the polycistronic RNA. | | | | | | | |
| **Table S2 (cont.)** Potential mRNA targets of the putative *Actinobacillus pleuropneumoniae* novel *trans*-acting RNAs described in this work. Putative binding positions in target mRNA 5’ UTR were determined using TargetRNA2 ([Kery et al. 2014](#_ENREF_34)). | | | | | | | |
| **RNA ID** | **Rank** | **Targetgene** | **Locus** | **Energy (kcal/mol)** | ***p*-value** | **Putative binding position in target mRNA*** | **Description** |
|  | 14 | *cca* | APL_0915 | -9.66 | 0.028 | -63 to -54 | multifunctional tRNA nucleotidyl transferase/2'3'-cyclic phosphodiesterase/2'nucleotidase/phosphatase |
|  | 15 | ***malQ*** | APL_1240 | -9.59 | 0.029 | -48 to -38 | 4-alpha-glucanotransferase |
|  | 16 | *lyx* | APL_1564 | -9.48 | 0.030 | -70 to -61 | L-xylulose kinase |
|  | 17 | *pyrG* | APL_0136 | -9.37 | 0.032 | -58 to -47 | CTP synthetase |
|  | 18 | *copA* | APL_1265 | -9.19 | 0.034 | -60 to -52 | copper-transporting P-type ATPase |
|  | 19 | *-* | APL_0271 | -8.58 | 0.044 | +1 to +10 | chelated iron transport system ATP-binding protein |
|  | 20 | *uspA* | APL_0655 | -8.56 | 0.045 | -29 to -13 | universal stress protein A-like protein |
|  | 21 | *lacZ* | APL_0997 | -8.52 | 0.045 | -42 to -32 | beta-galactosidase |
|  | 22 | *ostA* | APL_0962 | -8.5 | 0.046 | -55 to -44 | organic solvent tolerance protein |
|  | 23 | *dipZ* | APL_1359 | -8.42 | 0.047 | -18 to -1 | thiol:disulfide interchange protein |
|  | 24 | *pheT* | APL_0609 | -8.38 | 0.048 | -60 to -51 | phenylalanyl-tRNA synthetase subunit beta |
| **Arrc07** | 1 | *napF* | APL_1431 | -14.11 | 0.002 | -80 to -68 | ferredoxin-type protein NapF |
|  | 2 | *menD* | APL_1750 | -12.32 | 0.006 | +6 to +17 | 2-succinyl-5-enolpyruvyl-6-hydroxy-3-cyclohexene-1-carboxylate synthase |
|  | 3 | *pgaA* | APL_1921 | -11.71 | 0.009 | -80 to -72 | biofilm PGA synthesis protein PgaA |
|  | 4 | *rumB* | APL_1112 | -11.33 | 0.011 | -22 to -14 | 23S rRNA methyluridine methyltransferase |
|  | 5 | *focA* | APL_1037 | -10.1 | 0.022 | +4 to +12 | formate transporter |
|  | 6 | *mtfA* | APL_0685 | -10.07 | 0.023 | -26 to -12 | RNA 2'-O-ribose methyltransferase |
|  | 7 | *yedE* | APL_1977 | -10.02 | 0.023 | -64 to -50 | inner membrane protein |
|  | 8 | *cspC* | APL_0118 | -9.45 | 0.030 | -56 to -48 | cold shock-like protein CspC |
|  | 9 | *vacB* | APL_1478 | -9.2 | 0.034 | +7 to +19 | ribonuclease R |
|  | 10 | *pgsA* | APL_0275 | -9.18 | 0.034 | -30 to -16 | phosphatidylglycerophosphate synthase |
|  | 11 | *msbA* | APL_0778 | -9.16 | 0.035 | +12 to +20 | lipid transporter ATP-binding protein/permease |
|  | 12 | *grpE* | APL_0367 | -9.06 | 0.036 | -80 to -70 | heat shock protein GrpE |
|  | 13 | *mreC* | APL_0436 | -8.99 | 0.037 | -36 to -28 | rod shape-determining protein MreC |
|  | 14 | *purE* | APL_0659 | -8.72 | 0.042 | +11 to +12 | phosphoribosylaminoimidazole carboxylase catalytic subunit |
|  | 15 | *pepB* | APL_0388 | -8.55 | 0.045 | -64 to -55 | aminopeptidase B |
| **Arrc08** | 1 | *pta* | APL_0644 | -11.72 | 0.009 | +1 to +11 | phosphate acetyltransferase |
|  | 2 | *ung* | APL_0362 | -10.59 | 0.017 | +9 to +20 | uracil-DNA glycosylase |
|  | 3 | *lpxD* | APL_0409 | -10.26 | 0.021 | -42 to -28 | UDP-3-O-[3-hydroxymyristoyl] glucosamine N-acyltransferase |
|  | 4 | *sufI* | APL_1487 | -9.39 | 0.031 | +10 to +20 | protein SufI |
|  | 5 | *aroQ* | APL_1862 | -9.19 | 0.034 | +6 to +20 | 3-dehydroquinate dehydratase |
|  | 6 | *hyaB* | APL_1334 | -9.15 | 0.035 | -47 to -35 | hydrogenase 2 large subunit |
|  | 7 | *tmk* | APL_1817 | -8.93 | 0.038 | +4 to +16 | thymidylate kinase |
|  | 8 | *ftsY* | APL_1346 | -8.43 | 0.047 | +2 to +12 | cell division protein FtsY |
| **Arrc11** | 1 | *copA* | APL_1265 | -16 | 0.000 | -5 to -14 | copper-transporting P-type ATPase |
|  | 2 | ***murG*** | APL_0018 | -12.89 | 0.004 | -1 to -15 | undecaprenyldiphospho-muramoylpentapeptide beta-N-acetylglucosaminyltransferase |
|  | 3 | ***rpsK*** | APL_1782 | -11.59 | 0.010 | -1 to +15 | 30S ribosomal protein S11 |
|  | 4 | *ispH* | APL_1520 | -11.22 | 0.012 | -11 to +3 | 4-hydroxy-3-methylbut-2-enyl diphosphate reductase |
|  | 5 | *ccmF* | APL_1367 | -10.77 | 0.016 | -14 to -5 | cytochrome c-type biogenesis protein CcmF |
|  | 6 | *fdhE* | APL_0896 | -9.85 | 0.025 | -77 to -68 | formate dehydrogenase accessory protein FdhE |
|  | 7 | ***metN*** | APL_0912 | -9.83 | 0.025 | +4 to +14 | DL-methionine transporter ATP-binding subunit |
|  | 8 | *ung* | APL_0362 | -9.72 | 0.027 | +7 to +17 | uracil-DNA glycosylase |
|  | 9 | *dam* | APL_0192 | -9.02 | 0.037 | -80 to -68 | DNA adenine methylase |
|  | 10 | *ftsK* | APL_0618 | -8.84 | 0.040 | -80 to -66 | DNA translocase FtsK |
|  | 11 | *ulaG* | APL_1701 | -8.65 | 0.043 | -2 to +11 | L-ascorbate 6-phosphate lactonase |
|  | 12 | *djlA* | APL_0306 | -8.63 | 0.043 | +2 to +16 | Dna-J like membrane chaperone protein |
|  | 13 | *radA* | APL_0881 | -8.54 | 0.045 | -1 to 15 | DNA repair protein RadA |
|  | 14 | *apbE* | APL_0156 | -8.52 | 0.046 | -27 to -18 | thiamine biosynthesis lipoprotein ApbE |
|  | 15 | *eno* | APL_1113 | -8.48 | 0.046 | -1 to +15 | phosphopyruvate hydratase |
| *Position of target mRNA interaction with sRNA given in relation to +1 translation starting point, in accordance to the transcriptome of *Actinobacillus pleuropneumoniae* reference strain L20 (Genbank access NC_009053), considering the region around the 5’UTR of each mRNA. Target genes depicted in bold are located within operons and thus the putative region of interaction between the sRNA and the mRNA is in the interior of the polycistronic RNA. | | | | | | | |
| **Table S2 (cont.)** Potential mRNA targets of the putative *Actinobacillus pleuropneumoniae* novel *trans*-acting RNAs described in this work. Putative binding positions in target mRNA 5’ UTR were determined using TargetRNA2 ([Kery et al. 2014](#_ENREF_34)). | | | | | | | |
| **RNA ID** | **Rank** | **Targetgene** | **Locus** | **Energy (kcal/mol)** | ***p*-value** | **Putative binding position in target mRNA *** | **Description** |
| **Arrc14** | 1 | *proA* | APL_1951 | -15.29 | 0.001 | -79 to -63 | gamma-glutamyl phosphate reductase |
|  | 2 | *htpG* | APL_0987 | -13.19 | 0.003 | -77 to -63 | heat shock protein 90 |
|  | 3 | *hcp* | APL_1546 | -12.95 | 0.004 | +5 to +20 | hydroxylamine reductase |
|  | 4 | *ompP2A* | APL_0006 | -12.62 | 0.005 | +1 to +17 | outer membrane protein P2 |
|  | 5 | *fdx* | APL_0924 | -12.12 | 0.007 | +8 to +20 | 2Fe-2S ferredoxin |
|  | 6 | *cbiK* | APL_1624 | -11.88 | 0.008 | -80 to -69 | periplasmic binding protein CbiK |
|  | 7 | *mutT* | APL_0241 | -11.01 | 0.014 | +1 to +16 | mutator mutT protein |
|  | 8 | *grcA* | APL_0361 | -10.98 | 0.014 | -70 to -60 | autonomous glycyl radical cofactor GrcA |
|  | 9 | *ispZ* | APL_0972 | -10.95 | 0.014 | -80 to -66 | intracellular septation protein A |
|  | 10 | *bioD* | APL_0614 | -10.38 | 0.019 | -56 to -44 | dithiobiotin synthetase |
|  | 11 | *znuC* | APL_0456 | -10.12 | 0.022 | -54 to -42 | high-affinity zinc uptake system ATP-binding protein ZnuC |
|  | 12 | *glmS* | APL_1631 | -10.03 | 0.023 | -1 to +9 | glucosamine--fructose-6-phosphate aminotransferase |
|  | 13 | *galT* | APL_0994 | -9.92 | 0.024 | -53 to -40 | galactose-1-phosphate uridylyltransferase |
|  | 14 | *wecB* | APL_1552 | -9.84 | 0.025 | -73 to -58 | UDP-N-acetylglucosamine 2-epimerase |
|  | 15 | *rbsD* | APL_1669 | -9.82 | 0.026 | +1 to +16 | D-ribose pyranase |
|  | 16 | *frdA* | APL_1529 | -9.8 | 0.026 | +2 to +12 | fumarate reductase flavoprotein subunit |
|  | 17 | *tadD* | APL_0549 | -9.78 | 0.026 | -64 to -50 | tight adherence protein D |
|  | 18 | *zwf* | APL_1311 | -9.77 | 0.026 | -79 to -64 | glucose-6-phosphate 1-dehydrogenase |
|  | 19 | *glmU* | APL_0588 | -9.47 | 0.030 | -59 to -41 | bifunctional N-acetylglucosamine-1-phosphate uridyltransferase/glucosamine-1-phosphate acetyltransferase |
|  | 20 | *nrfX* | APL_1051 | -9.37 | 0.032 | +1 to +14 | disulfide bound formation protein DsbE |
|  | 21 | *leuS* | APL_0872 | -9.07 | 0.036 | -61 to -50 | leucyl-tRNA synthetase |
|  | 22 | *ompP2* | APL_0649 | -9.03 | 0.037 | +1 to +14 | Outer membrane protein P2 precursor (OMP P2) |
|  | 23 | *tadE* | APL_0548 | -8.76 | 0.041 | +8 to +20 | tight adherence protein E |
|  | 24 | *torD* | APL_1797 | -8.51 | 0.046 | -80 to -66 | chaperone protein TorD |
|  | 25 | *afuB_2* | APL_0564 | -8.51 | 0.046 | +9 to +19 | ferric transport system permease protein |
|  | 26 | *mglB* | APL_1420 | -8.32 | 0.049 | +1 to +17 | D-galactose-binding periplasmic protein |
| **Arrc17** | 1 | ***rpsM*** | APL_1781 | -15.9 | 0.000 | -46 to -27 | 30S ribosomal protein S13 |
|  | 2 | *arsR* | APL_1090 | -14.2 | 0.001 | -80 to -63 | transcriptional regulator |
|  | 3 | *menC* | APL_0353 | -12.1 | 0.007 | -79 to -65 | O-succinylbenzoate synthase |
|  | 4 | *rsmB* | APL_1560 | -11.75 | 0.009 | -76 to -58 | ribosomal RNA small subunit methyltransferase B |
|  | 5 | *fdhE* | APL_0896 | -11.47 | 0.010 | +1 to +14 | formate dehydrogenase accessory protein FdhE |
|  | 6 | *frdB* | APL_1528 | -11.3 | 0.012 | -18 to -15 | fumarate reductase iron-sulfur subunit |
|  | 7 | *cysW* | APL_1847 | -10.12 | 0.022 | +1 to +15 | sulfate transport system permease protein cysW |
|  | 8 | *dppD* | APL_0067 | -9.58 | 0.029 | -23 to -15 | dipeptide transporter ATP-binding subunit |
|  | 9 | *coaE* | APL_0876 | -9.49 | 0.030 | -66 to -56 | dephospho-CoA kinase |
|  | 10 | *priA* | APL_1032 | -9.28 | 0.033 | -65 to -48 | primosome assembly protein PriA |
|  | 11 | *rnfB* | APL_0166 | -9.18 | 0.034 | +1 to +19 | electron transport complex protein RnfB |
|  | 12 | *pbpB* | APL_1823 | -9.16 | 0.035 | -20 to -1 | penicillin-binding protein 1B (PBP1b) |
|  | 13 | *purE* | APL_0659 | -8.77 | 0.041 | +12 to +20 | phosphoribosylaminoimidazole carboxylase catalytic subunit |
|  | 14 | *rnfA* | APL_0165 | -8.7 | 0.042 | -64 to -53 | Na(+)-translocating NADH-quinone reductase subunit E |
|  | 15 | *thrA* | APL_0250 | -8.5 | 0.046 | -39 to -26 | bifunctional aspartokinase I/homoserine dehydrogenase I |
|  | 16 | *dksA* | APL_0175 | -8.39 | 0.048 | -13 to -5 | DnaK suppressor protein |
| **Arrc20** | 1 | *pmbA* | APL_0729 | -13.03 | 0.004 | -45 to -34 | antibiotic maturation factor |
|  | 2 | *-* | APL_1678 | -11.76 | 0.009 | -35 to -23 | ferredoxin |
|  | 3 | *corA* | APL_1981 | -10.53 | 0.018 | -44 to -30 | magnesium/nickel/cobalt transporter CorA |
|  | 4 | *pta* | APL_0644 | -9.64 | 0.028 | -3 to +9 | phosphate acetyltransferase |
|  | 5 | *fldA* | APL_1219 | -9.28 | 0.033 | -79 to -66 | flavodoxin FldA |
|  | 6 | *lctP* | APL_0447 | -9.14 | 0.035 | +5 to +19 | L-lactate permease |
|  | 7 | *ureA* | APL_1618 | -8.47 | 0.046 | -79 to -61 | urease subunit gamma |
|  | 8 | *fumC* | APL_1757 | -8.32 | 0.049 | +6 to +17 | fumarate hydratase |
|  |  |  |  |  |  |  |  |
| *Position of target mRNA interaction with sRNA given in relation to +1 translation starting point, in accordance to the transcriptome of *Actinobacillus pleuropneumoniae* reference strain L20 (Genbank access NC_009053), considering the region around the 5’UTR of each mRNA. Target genes depicted in bold are located within operons and thus the putative region of interaction between the sRNA and the mRNA is in the interior of the polycistronic RNA. | | | | | | | |
| **Table S2 (cont.)** Potential mRNA targets of the putative *Actinobacillus pleuropneumoniae* novel *trans*-acting RNAs described in this work. Putative binding positions in target mRNA 5’ UTR were determined using TargetRNA2 ([Kery et al. 2014](#_ENREF_34)). | | | | | | | |
| **RNA ID** | **Rank** | **Targetgene** | **Locus** | **Energy (kcal/mol)** | ***p*-value** | **Putative binding position in target mRNA *** | **Description** |
| **Arrc21** | 1 | ***ubiC*** | APL_1840 | -11.02 | 0.014 | -74 to -56 | 4-hydroxybenzoate synthetase |
|  | 2 | *apxIIA* | APL_0956 | -10.73 | 0.016 | -51 to -43 | RTX-II toxin determinant A |
|  | 3 | *bioA* | APL_0942 | -10.26 | 0.020 | +1 to +17 | adenosylmethionine-8-amino-7-oxononanoate aminotransferase |
|  | 4 | *anmK* | APL_1535 | -9.57 | 0.029 | +12 to +20 | anhydro-N-acetylmuramic acid kinase |
|  | 5 | *cysK* | APL_0248 | -9.18 | 0.034 | -10 to +6 | cysteine synthase |
|  | 6 | *guaA* | APL_0592 | -9.08 | 0.036 | -11 to +8 | GMP synthase |
|  | 7 | *malE* | APL_1237 | -8.6 | 0.044 | -59 to -50 | maltose ABC transporter periplasmic protein |
|  | 8 | *menA* | APL_1461 | -8.49 | 0.046 | -80 to -67 | 1,4-dihydroxy-2-naphthoate octaprenyltransferase |
|  | 9 | *coaE* | APL_0876 | -8.3 | 0.050 | -6 to +9 | dephospho-CoA kinase |
| *Position of target mRNA interaction with sRNA given in relation to +1 translation starting point, in accordance to the transcriptome of *Actinobacillus pleuropneumoniae* reference strain L20 (Genbank access NC_009053), considering the region around the 5’UTR of each mRNA. Target genes depicted in bold are located within operons and thus the putative region of interaction between the sRNA and the mRNA is in the interior of the polycistronic RNA. | | | | | | | |

| **Table S3** Distribution of the small RNA candidates described in this work among *Actinobacillus pleuropneumoniae* strains and clinical isolates and other *Pasteurellaceae.* | | | | | | | | | | | | | | | | | | | | | | | |
| --- | --- | --- | --- | --- | --- | --- | --- | --- | --- | --- | --- | --- | --- | --- | --- | --- | --- | --- | --- | --- | --- | --- | --- |
|  | **RNAs** | | | | | | | | | | | | | | | | | | | | | | |
| **Species** | **Arrc01** | **Arrc02** | **Arrc03** | **Arrc04** | **Arrc05** | **Arrc06** | **Arrc07** | **Arrc08** | **Arrc09** | **Arrc10** | **Arrc11** | **Arrc12** | **Arrc13** | **Arrc14** | **Arrc15** | **Arrc16** | **Arrc17** | **Arrc18** | **Arrc19** | **Arrc20** | **Arrc21** | **Arrc22** | **Arrc23** |
| ***Actinobacillus pleuropneumoniae*** | | | | | | | | | | | | | | | | | | | | | | | |
| Shope4074 | x | x | x | x | x | x | x | x | x | x | x | x | x | x | x | x | x | x | x | x | x | x | x |
| S1536 | x | x | x | x | x | x | x | x | x | x | x | x | x | x | x | x | x | x | x | x | x | x | x |
| JL03 | x | x | x | x | x | x | x | x | x | x | x | x | x | x | x | x | x | x | x | x | x | x | x |
| M62 | x | x | x | x | x | x | x | x | x | x | x | x | x | x | x | x | x | x | x | x | x | x | x |
| L20 | x | x | x | x | x | x | x | x | x | x | x | x | x | x | x | x | x | x | x | x | x | x | x |
| Femo | x | x | x | x | x | x | x | x | x | x | x | x | x | x | x | x | x | x | x | x | x | x | x |
| AP76 | x | x | x | x | x | x | x | x | x | x | x | x | x | x | x | x | x | x | x | x | x | x | x |
| CVJ13261 | x | x | x | x | x | x | x | x | x | x | x | x | x | x | x | x | x | x | x | x | x | x | x |
| D13039 | x | x | x | x | x | x | x | x | x | x | x | x | x | x | x | x | x | x | x | x | x | x | x |
| 56153 | x | x | x | x | x | x | x | x | x | x | x | x | x | x | x | x | x | x | x | x | x | x | x |
| 1096 | x | x | x | x | x | x | x | x | x | x | x | x | x | x | x | x | x | x | x | x | x | x | x |
| N273 | x | - | x | x | x | x | x | x | x | x | x | x | x | x | x | x | x | x | x | x | x | x | x |
| 518 | x | x | x | x | x | x | x | x | x | x | x | x | x | x | x | x | x | x | x | x | x | x | x |
| 5651 | x | x | x | x | x | x | x | x | x | x | x | x | x | x | x | x | x | x | x | x | x | x | x |
| 597 | x | x | x | x | x | x | x | x | x | x | x | x | x | x | x | x | x | x | x | x | x | x | x |
| 780 | x | x | x | x | x | x | x | x | x | x | x | x | x | x | x | x | x | x | x | x | x | x | x |
| 460 | x | x | x | x | x | x | x | x | x | x | x | x | x | x | x | x | x | x | x | x | x | x | x |
| 1022 | x | x | x | x | x | x | x | x | x | x | x | x | x | x | x | x | x | x | x | x | x | x | x |
| ***Actinobacillus succinogenes*** | | | | | | | | | | | | | | | | | | | | | | | |
| 130Z | - | - | - | - | - | - | - | - | - | - | - | x | - | - | x | - | x | - | - | - | x | - | x |
| ***Actinobacillus suis*** |  |  |  |  |  |  |  |  |  |  |  |  |  |  |  |  |  |  |  |  |  |  |  |
| H91-0380 | x | x | x | x | x | x | x | x | x | x | x | x | x | x | x | x | x | x | x | x | x | x | x |
| ***Aggregatibacter actinomycetemcomitans*** | | | | | | | | | | | | | | | | | | | | | | | |
| ANH9381 | - | - | - | - | - | - | - | - | - | - | - | - | - | - | x | - | - | - | - | - | x | - | x |
| D11S-1 | - | - | - | - | - | - | - | - | - | - | - | - | - | - | x | - | - | - | - | - | x | - | x |
| D7S-1 | - | - | - | - | - | - | - | - | - | - | - | - | - | - | x | - | - | - | - | - | x | - | x |
| ***Aggregatibacter aphrophilus*** | | | | | | | | | | | | | | | | | | | | | | | |
| NJ8700 | - | - | - | - | - | - | - | - | - | - | - | - | - | - | x | - | - | - | - | - | x | - | x |
| ***Gallibacterium anatis*** |  |  |  |  |  |  |  |  |  |  |  |  |  |  |  |  |  |  |  |  |  |  |  |
| UMN179 | - | - | - | - | - | - | - | - | - | - | - | x | - | - | x | - | x | - | - | - | x | - | x |
| ***Haemophilus ducrey*** |  |  |  |  |  |  |  |  |  |  |  |  |  |  |  |  |  |  |  |  |  |  |  |
| 35000HP | - | x | - | - | - | - | x | x | - | - | - | x | x | - | x | x | x | - | - | - | x | - | x |
| ***Haemophilus influenzae*** |  |  |  |  |  |  |  |  |  |  |  |  |  |  |  |  |  |  |  |  |  |  |  |
| 86-028NP | - | - | - | - | - | - | - | - | x | - | - | - | - | - | x | - | x | - | - | - | x | - | x |
| 10810 | - | - | - | - | - | - | - | - | x | - | - | - | - | - | x | - | x | - | - | - | x | - | x |
| F3031 | - | - | - | - | - | - | - | - | x | - | - | - | - | - | x | - | x | - | - | - | x | - | x |
| F3047 | - | - | - | - | - | - | - | - | x | - | - | - | - | - | x | - | x | - | - | - | x | - | x |
| PittEE | - | - | - | - | - | - | - | - | x | - | - | - | - | - | x | - | x | - | - | - | x | - | x |
| PittGG | - | - | - | - | - | - | - | - | x | - | - | - | - | - | x | - | x | - | - | - | x | - | x |
| R2864 | - | - | - | - | - | - | - | - | x | - | - | - | - | - | x | - | x | - | - | - | x | - | x |
| R2866 | - | - | - | - | - | - | - | - | x | - | - | - | - | - | x | - | x | - | - | - | x | - | x |
| RdKW20 | - | - | - | - | - | - | - | - | x | - | - | - | - | - | x | - | x | - | - | - | x | - | x |
| ***Haemophilus parainfluenzae*** | | | | | | | | | | | | | | | | | | | | | | | |
| T3T1 | - | - | - | - | - | - | x | - | - | - | - | - | - | - | x | - | - | - | - | - | - | - | x |
|  |  |  |  |  |  |  |  |  |  |  |  |  |  |  |  |  |  |  |  |  |  |  |  |
|  |  |  |  |  |  |  |  |  |  |  |  |  |  |  |  |  |  |  |  |  |  |  |  |
| **Table S3 (cont.)** Distribution of the small RNA candidates described in this work among *Actinobacillus pleuropneumoniae* strains and clinical isolates and other *Pasteurellaceae***.* | | | | | | | | | | | | | | | | | | | | | | | |
|  | **RNAs** | | | | | | | | | | | | | | | | | | | | | | |
| **Species** | **Arrc01** | **Arrc02** | **Arrc03** | **Arrc04** | **Arrc05** | **Arrc06** | **Arrc07** | **Arrc08** | **Arrc09** | **Arrc10** | **Arrc11** | **Arrc12** | **Arrc13** | **Arrc14** | **Arrc15** | **Arrc16** | **Arrc17** | **Arrc18** | **Arrc19** | **Arrc20** | **Arrc21** | **Arrc22** | **Arrc23** |
| ***Haemophilus parasuis*** |  |  |  |  |  |  |  |  |  |  |  |  |  |  |  |  |  |  |  |  |  |  |  |
| SH0165 | x | - | - | - | - | - | x | - | - | - | - | - | x | - | x | - | x | - | - | - | x | - | x |
| ***Haemophilus somnus*** |  |  |  |  |  |  |  |  |  |  |  |  |  |  |  |  |  |  |  |  |  |  |  |
| 129T | - | - | - | - | - | - | - | - | - | - | - | - | - | - | x | - | x | - | - | - | x | - | x |
| 2336 | - | - | - | - | - | - | - | - | - | - | - | - | - | - | x | - | x | - | - | - | x | - | x |
| ***Mannheimia haemolytica*** |  |  |  |  |  |  |  |  |  |  |  |  |  |  |  |  |  |  |  |  |  |  |  |
| M42548 | - | - | x | - | - | - | x | x | - | x | - | - | x | - | x | x | x | - | - | x | x | - | x |
| D153 | - | - | x | - | - | - | x | x | - | x | - | - | x | - | x | x | x | - | - | x | x | - | x |
| D171 | - | - | x | - | - | - | x | x | - | x | - | - | x | - | x | x | x | - | - | x | x | - | x |
| D174 | - | - | x | - | - | - | x | x | - | x | - | - | x | - | x | x | x | - | - | x | x | - | x |
| USDA-ARS-USMARC-183 | - | - | x | - | - | - | x | x | - | x | - | - | x | - | x | x | x | - | - | x | x | - | x |
| ***Mannheimia succiniciproducens*** | | | | | | | | | | | | | | | | | | | | | | | |
| MBEL55E | - | - | x | - | - | x | - | - | - | - | - | x | - | - | x | - | - | - | - | - | x | - | x |
| ***Mannheimia varigena*** |  |  |  |  |  |  |  |  |  |  |  |  |  |  |  |  |  |  |  |  |  |  |  |
| USDA-ARS-USMARC-1261 | - | - | - | - | - | x | x | x | - | x | - | - | x | - | x | x | x | - | - | x | x | - | x |
| USDA-ARS-USMARC-1296 | - | - | x | - | - | x | x | x | - | x | - | - | x | - | x | x | x | - | - | x | x | - | x |
| USDA-ARS-USMARC-1312 | - | - | x | - | - | x | x | x | - | x | - | - | x | - | x | x | x | - | - | x | x | - | x |
| USDA-ARS-USMARC-1388 | - | - | x | - | - | - | x | x | - | x | - | - | x | - | x | x | x | - | - | x | x | - | x |
| ***Pasteurella multocida*** |  |  |  |  |  |  |  |  |  |  |  |  |  |  |  |  |  |  |  |  |  |  |  |
| Pm70 | - | - | - | - | - | - | - | - | - | - | - | - | - | - | x | - | - | - | - | - | x | - | x |
| HN06 | - | - | - | - | - | - | - | - | - | - | - | - | - | - | x | - | - | - | - | - | x | - | x |
| * “x” denotes the presence of a determined RNA, while “-” denotes the absence. | | | | | | | | | | | | | | | | | | | | | | | |
|  |  |  |  |  |  |  |  |  |  |  |  |  |  |  |  |  |  |  |  |  |  |  |  |
|  |  |  |  |  |  |  |  |  |  |  |  |  |  |  |  |  |  |  |  |  |  |  |  |
|  |  |  |  |  |  |  |  |  |  |  |  |  |  |  |  |  |  |  |  |  |  |  |  |
|  |  |  |  |  |  |  |  |  |  |  |  |  |  |  |  |  |  |  |  |  |  |  |  |

| **Table S4.** Genbank accession numbers of the *Pasteurellaceae* genomes analyzed in this work. | |
| --- | --- |
| **Species** | **Strain (Genbank accession number)** |
| *Actinobacillus pleuropneumoniae* | Reference strains: Shope4074, serotype 1 (ADOD00000000); S1536, serotype 2 (ADOE00000000); JL03, serotype 3 (NC_010278); M62, serotype 4 (ADOF00000000); L20, serotype 5 (NC_009053); Femo, serotype 6 (ADOG00000000); AP76, serotype 7 (NC_010939); CVJ13261, serotype 9 (ADOI00000000); D13039, serotype 10 (ADOJ00000000); 56153, serotype 11 (ADOK00000000); 1096, serotype 12 (ADOL00000000); N273, serotype 13 (ADOM00000000).  Clinical isolates: 518 (JSVZ00000000), 5651 (JSVY00000000), 597 (JSVX00000000), 780 (JSVV00000000), 460 (JSVG00000000), 1022 (JSVF00000000), all serotype 8. |
| *Actinobacillus succinogenes* | 130Z (NC_009655) |
| *Actinobacillus suis* | H91-0380 (NC_018690) |
| *Aggregatibacter actinomycetemcomitans* | ANH9381 (CP003099), D11S-1 (CP001733), D7S-1 (CP003496) |
| *Aggregatibacter aphrophilus* | NJ8700 (CP001607) |
| *Gallibacterium anatis* | UMN179 (NC_015460) |
| *Haemophilus ducreyi* | 35000HP (NC_002940) |
| *Haemophilus influenzae* | RdKW20 (NC_000907), 10810 (NC_016809), F3047 (NC_014922), 86-028NP (NC_007146), F3031 (NC_014920), PittGG (CP000672), PittEE (CP000671), R2846 (CP002276), R2866 (CP002277) |
| *Haemophilus parainfluenzae* | T3T1 (NC_015964) |
| *Haemophilus parasuis* | SH0165 (NC_011852) |
| *Haemophilus somnus* | 129T (NC_008309), 2336 (NC_010519) |
| *Mannheimia haemolytica* | M42548 (NC_021082), D153 (NC_021743), D171 (NC_021738), D174 (NC_021739), USDA-ARS-USMARC-183 (NC_020833) |
| *Mannheimia succiniciproducens* | MBEL55E (NC_006300) |
| *Mannheimia varigena* | USDA-ARS-USMARC-1261 (CP006942); USDA-ARS-USMARC-1296 (CP006943); USDA-ARS-USMARC-1312 (CP006944); USDA-ARS-USMARC-1388 (CP006953) |
| *Pasteurella multocida* | Pm70 (NC_002663), HN06 (NC_017027) |

1. **FMN Riboswitch – Rfam Accession RF00050**

SS. ::((((((((,,,,,,<<<_______>>>,,,,,<<<<___________________>>>

FMN-Rfam AAUCGUCCUCAGGGCAGGGUGAAAUUCCCUACCGGCGGUAAUUAAUUAAAUUUAAGCCCG

FMN-App AAUAAUCUUCAGGGCAGGGUGAAAUUCCCGAUCGGCGGUAA-------------AGUCCG

***..** ********************* * ********* ** ***

SS. >,,,,,,,,,,,,,,,,,,,,,,,,,,,,<<<<<_______>>>>>,,,,,<<<______

FMN-Rfam CGAGCGUUUAAAAUUUUAAAGUCAGCAGAUCCGGUGAAAUUCCGGAGCCGACGGUAUAGU

FMN-App CGAGCCGAACGA-AAAAGGUUUGGCAGGAACCGGUGAGAUUCCGGUACCGACAGUAUAGU

***** ..* .. * . ..** *******.******* .*****.*******

SS. >>>,,,,,,,))))))))::

FMN-Rfam CCGGAUGGAAGAGGACGAAA

FMN-App CUGGAUGGAAGAAGAUGAAA

* **********.** ****

1. **His Attenuator – Rfam Accession RF00514**

SS. :::::::<<<<<<<.--<<<<<________>>>>>-.>>>>-.->>>.<<<<<<<<___

His-Rfam UCAUCACCACCAUC-AUCCUGACUAGUCUUUCAGG-AGAUGU-UGUG-CUGGAAGGCAA

His-App UCAUCACCAUCAUUCAAGCUGA--UAUCUUUCGGGCAUUUGAGUGUGGUCGGAAGAUAG

********* *** * **** .******.** *. ** **** ***** *

SS. _____>>>>>>>>--....................,,,,,,,,,,,,,,,,,<<<.<<<

His-Rfam UUAUACCUUCCAGAG--------------------UGAUAUAAAAACAAAUUAAA-AAA

His-App AAA-AUCUUCCGAGUACGUAUAAAGCAAAAUCGCA---UAUCGCAGCAAAAUACACAAC

* * *****... *** . *.**** ** * **

SS. <<<<<<<<<<_____>>>>>>>>>>>>>>>>:::

His-Rfam CCCUCGGGAGAUCAUCUCCCGAGGGUUUUUUUUU

His-App CUCUCGGAAG-GCAACUUUCGAGAGGUUUUUUAU

* *****.** ** ** ****.* ****** *

**Figure S1**. Nucleotide sequence alignments and secondary structures (SS) of the *cis-*acting sRNAs whose expressions were confirmed in this work. Alignments were made between the element predicted for *Actinobacillus pleuropneumoniae* (App) and the consensus sequence available from the Rfam database. Figure shows alignments for both the FMN riboswitch (A) and the Histidine attenuator (B). Sequential “ **<** and **>** ” represent sequences that pair with each other, separated by loops, represented by “**____**”. Regions not involved with secondary structures are represented by “ **,** ” when in the interior of the sequence or by “ **:** ” when in the extremities. Distant regions that pair with each other are represented by “ **(** and **)** ”. Expression platforms are highlighted in yellow.

**Figure S2 (Pages 10 to 15).** Sequence alignment of the novel *trans*-acting sRNAs described in this work. *Pasteurellaceae* analyzed: *Actinobacillus pleuropneumoniae* (App), *A. succinogenes* (Asc), *A. suis* (Asu), *Aggregatibacter actinomycetemcomitans* (Agc), *Aggregatibacter aphrophilus* (Agp), *Gallibacterium anatis* (Gan), *Haemophilus ducreyi* (Hdu), *H. influenzae* (Hin), *H. parainfluenzae* (Hpa), *H. parasuis* (Hps). *H. somuns* (Hsm), *Mannheimia haemolytica* (Mha), *M. succiniproducens* (Msc), *M. varigena* (Mva), *Pasteurella multocida* (Pmu). Regions containing the promoter region (-35 and -10), the sRNA coding strand, and putative transcription terminators are highlighted.

**ARRC01**

**-35 -10**

App TAAACGGAATGTGACTACATTATTTTTTTTGATAGATAAAATTAAATTTTTTAGTTTGTC

Asu ----------CATTATTTTTTTTGATAGTTAAAATTAAAATTTTTAGCT-------TGTC

Hps --ATTACTTTTTTTGATAAATAAAATTAAAAA----------------ATCTCGTTTGTA

* * * ***

**sRNA coding sequence**

App ATACTGGTCGAACATCACTATAATGC-GCGCCGTACTTAGACGGATAGTGATAGCTTAAG

Asu ATACTGGTCTAACATCACTATAATGC-GCGCCGTACTTAGACGGATAGTGATAGTTAAGC

Hps TAACTGGTCAAACATCTCTAAAATTCAGCACCATACTTAGATGGATAGTGA---------

******* ****** *** *** * ** ** ******** *********

App TTTCCGAAT---TATTACGGTAAATTCAGCTATTTCAGATTTTTAAGTATGATGTTGTGT

Asu AGTAAGAATGTATTTTTTTACTGATTTAACTATTTCAGATTTTTAAGTATGATGTTGTGT

Hps ---------------TAGCTGTCTTTTAGCTATTTTTGAATTTTAAGTATGATGTTGTGT

* ** * ****** ** ********************

**putative transcription terminator**

App TTGCATATTGGTCTAGGAAACTAGACTGGAGTAACATCA-AGTTACTCGTTTCACTTCCT

Asu TTGCATATTGGTCTAGGAAACTAGACTAGAGTAACATTT-AGTTACTCGTTTCACTTCCT

Hps TTGCATATTGGTCTAGGAAACTAGACTAGAGTAACAAAAAAGTTACTCGTTTCACTTCCT

*************************** ******** ********************

App GTATATTTTGAACCTTTTTGGTTTATAACCGTCCATTTTGGACGGTTTTTTTCGT--

Asu GTATATTTTGAACCCTTTTGGTTTATTGACCGCCCGATTTGGGCGGTTTTTTTTCGT

Hps GTATATTTTGAACCTTTTGGTTTAGCTACCGCCCATGTTGGGCGGTTTTTT------

************** *** * ** * ** ** * * *****

**ARRC02**

**-35 -10**

App CAGGTTGTTCTTGTTGTATTTCGGCTGTTTGTTCTAATTGCGCTTCGTTTGCAACTTCAA

Asu CAGGTTGTTCTTGTTGTACTTCGGCTGTTTGTTCTAATTGCGCTTCGTTTGCAACTTCAA

Hdu ------------------------------------------------------------

**sRNA coding sequence**

App CTTGTTCGGCTTGAGCTTGTGCATTTTCGTTTTGAGCTGTCATTTTAAATCCTCTT----

Asu CTTGTTCGGCTTGAGCTTGTGCATTTTCGTTTTGAGCTGTCATTTTAAATCCTCTT----

Hdu ------------------------TTTCTTTTTCAGTGGTC-ATATTAATCCTCTTGGTT

**** **** ** *** * * *********

App --CGTTTCAAATATATAAATGAAAAATCATTACTAATATAGCGTTAAATTTGCTGATTTC

Asu --CGTTTCAAATATATAAATGAAAAATCATTACTAATATAGCGTTAAATTTGCTGATTTC

Hdu TCAATTTATTAATTATTAATGAAGAATCGCCATTAATATGGCGACAGATTTTGTGATTTC

*** * *** ****** **** * ****** *** * **** *******

App AAGGTAAAAGCGGTAATGTGA-ATAAATTATTAAAAATAATTTGCAAAAAATTGCCGTTT

Asu AAGGTAAAAGCGGTAATGTGA-ATAAATTATTAAAAATAATTTGCAAAAAATTGCCGTTT

Hdu AAGGTTAAAATAGTGATGCTTATCAGATTATTAAAAAATAATCAGTAAAGTTTTTTATTT

***** *** ** *** * *********** * * *** ** ***

**putative transcription terminator**

App TGATTAGAAAATCCTTGCAAAATCTAGGGGGTGGCTTAAAATCGCCCTCGTCTTTTAAGC

Asu TGATTAGAAAATCCTTGCAAAATCTAGGGGGTGGCTTAAAATCGCCCTCGTCTTTTAAGC

Hdu TGGCTAGAAAAGGCTTGCAAAATATAAGGAGTGGCTTAGAATTTGCCTTGCTTTTTAGGC

** ******* ********** ** ** ******** *** *** * ***** **

**putative transcription terminator**

App CCTTAGGCAAGGCTATATTTTTTACTCATCTTTTCTTTAACGGAGAAAACTTAAATGAAA

Asu CCTTAGGCAAGGCTATATTTTTTACTCATCTTTTCTTTAACGGAGAAAACTTAAATGAAA

Hdu CTTTAGGCAAGGCTATATTTTTTAATCATCTTTTCTTTAACGGAGAAAACCTAAATGAAA

* ********************** ************************* *********

App AAATTAGCGGGTTTATTTGCAGCAGGTTTAGCGACAGTTGCAT

Asu AAATTAGCGGGTTTATTTGCAGCAGGTTTAGCGACAGTTGCAT

Hdu AAATTAGCGGGTTTATTTGCAGCAAGTTTAGTAACCGTTGCAT

************************ ****** ** *******

**ARRC04**

**-35 -10**

App AAAATGCGGATTGAAATCCGCCGAGCGTTAAGATTATTCAAATATCCGCCTGATAAACAA

Asu AAAATGCGAATTGAAATCCGCCGAGCGTTAAGATTATTCAAATATCCGCCCGATAAACAA

******** ***************************************** *********

App GCAGAAGCAGTCGAGTTTGTATTAAAACAGGCGGAAGTGATTGCAGATGAATTGAGTTCT

Asu GTGGAAGCAGTTGAGTTTGTATTAAGACAAGCGGAAGTGATTGCCGATGAATTAAGTTCA

* ******** ************* *** ************** ******** *****

**sRNA coding sequence**

App CCCTAATTTTGTTGTTTTGTTCATCAAATGAAAATAAATTCGCAAAAAGTGCTTGCATTG

Asu CCTTAGTGTTG---CTTTGTTAACCAAACAAAAACAAATTTGAAAAAAGTGCTTGCATTG

** ** * *** ****** * **** **** ***** * *****************

App GTTTTGGAAATCTCTATAATAAGCCACATCAGACGCGGGGTGGAGCAGCTTGGTAGCTCG

Asu GTTTTGGAAATCTCTATAATGAGCCACATCAGACGCGGGGTGGAGCAGCTTGGTAGCTCG

******************** ***************************************

App TCGGGCTCATAACCCGAAGGTCGTTGGTTCAAATCCGGCCCCCGCAACCAGTTTTAAGGC

Asu TCGGGCTCATAACCCGAAGGTCGTTGGTTCAAATCCGGCCCCCGCAACCAGTTTTAAGGC

************************************************************

**putative transcription terminator**

App TCACAGTTTTGTTTAAAACTGTGAGTTTTGTTTTATATAGCCTTTGTTAATTT

Asu TCACAGTTTTGATTAAAACTGTGAGTTTTGTTTTATATAGCCTTTGCTAATTT

*********** ********************************** ******

**ARRC05**

**-35 -10**

App CCGTACTTCAATGACGGCGGACGATGTACGTGCGGTCACTATCTATCTACAACGTAACGC

Asu CCGTACTTCAATGTCAGCAGATGATGTACGTGCGGTAACTATCTATCTACAACGTAACGC

************* * ** ** ************** ***********************

**sRNA coding sequence**

App AAAAGATGTTGCGGGTAGTTCACACTAATTTTATTTAACTTACAGGATACCGAGCTTGTA

Asu AAAAGATGTTGCGGGTAGTTCACACTAATTTTATTTAACTTACATGATACCGAGCTTGTA

******************************************** ***************

App TGCCTAAGTCTTAGATAAGGTGTGCAAGCCTGTGGCAAGCGGTTAAAATTCACAAAATTT

Asu TGCCTAAGTCTTAGATAAGGTGTGCAAGCCTGTGGCAAGCGGTCAAAATTCACAAAATTT

******************************************* ****************

**putative transcription terminator**

App TTGCAAAATCAGACCGCTT-ACACACCGGATAGTAAAGAAATGCACTATCCCCTCGTGTT

Asu TTGCAAAATCAGACCGCTTATCACACCGGATAGTAAAGAAATGCGCTATCCCCTCGTGTT

******************* *********************** ***************

App TAGAAAGGTGTCAAATTCAACTTAACTTTCAGAGGGTTATGCA

Asu TAGAAAGGTGTCAAATTTAACTTAACTTTCAGAGGGTTATGCA

***************** *************************

**ARRC07**

**-35 -10**

App TAAACATAACTTACAAGGAAAGAAAAGCAGGCTATAAGCGGTCGAATTTCGCCAAAAATT

Asu ------------------------------------------------------------

Hdu ------------------------------------------------------------

Hps ------------------------------------------------------------

Mva ------------------------------------------------------------

Mha ------------------------------------------------------------

**sRNA coding sequence**

App TGCAAAATCCTAAACACAAATAGAAAATAGGTTGTACTTTTCTCCCCTGTCCTTTTGCCT

Asu -----------------------------------ACTTTTCTCCCCTGTCCTTTTGCCT

Hdu -----------------------------------ACTTTTCTCCCCTGTCCTTTTGCCT

Hps ----------------------------------TACTTTTCTCCCCTGTCCTTTTGCCT

Mva -----------------------------------ACTTTTCTCCCCTGTCCTTTTGCCT

Mha -----------------------------------ACTTTTCTCCCCTGTCCTTTTGCCT

*************************

App GAGCGTTTCATGCTTACGCACT-------TGCGCCTTCGGCGTCCATTTTCCAT-TAACT

Asu GAGCGTTTCATGCATTCGCACT-------TGCGCCTTCGGCGTCCATTTTCCAT-T---T

Hdu GAGCGTTTCACATAAATTTGCTTTATATTTGCGCCTTCGGCGTCCATTTTCTGTACTATA

Hps GAGCGTTTCGCATTCATTGCTTT--------CGCCTTCGGCGTCCATTTT-----CTGCT

Mva GAGCGTTTCACACATTGCTGCATT-----TTCGCCTTCGGCGGCT-------------GA

Mha GAGCGTTTCACACATTGCTGTATT-----TTCGCCTTCGGCGGCG-------------GA

********* *********** *

App ACATGGATTGGATCTCTCCAAGGGTTCGTCCAGTAACTGTCCTCGCTATTGCTAGC----

Asu ACATGGATTGGATCTCTCCAAGGGTTCGTCCAGTAACTGTCCTCGCTATCGCTAGC----

Hdu GTACAGAGTGGATCTCTCCAAGGGTTCGTCCAGTAACTGTCCTCACATAGTTAAACTATT

Hps ATGCAGAGTGGATCTCTCCAAGGGTTC--------------GTCCAATAGCTGTCCTCGC

Mva TTTTTCATCAGCTCTCTCCAAGGGTTCGTCCAGTAACAGTC-CTCGCAACTTAGTT----

Mha TTTTA-ATCCGCTCTCTCCAAGGGTTCGTCCAGTAACAGTCCCCGCAGTGTTCACT----

* * ***************

**putative transcription terminator**

App ---------ACCTGAAAGATTTTACCTCGTCGGTAGAGCTTATCGCTCTTCTCCAGCTAC

Asu ---------ACCTGAAAGATTTTACCTCGTCGGTAGAGTTTTCACTCTTCTCCAGCTACC

Hdu GCACCTGAAAGATTTTACCTCGTCGGTAGAGCCTAAACTCTTCT-------CCAGCTACC

Hps TGTCGCACCTGAAAGATT---TTACCTCGTCGGTGTAGGGTTAT-------TCCCCACTC

Mva -------GCACCTGAAAGAGTTTACCTCGTCGGTGAAATGTTAATTCATTTCTCTCCAGC

Mha -------GCACCTGAAAGAGTTTACCTCGTCGGTGAAATGTTAATTCATTTCTCTCCAGC

* * * * * * *

App CTTCATCCGAACGTATCTTTT

Asu TTCATCCGAAC----------

Hdu TTCATCCGAACGTTTTCTTTT

Hps TCCAGCTATCTTCATCCGAAC

Mva TACCTTCATCCGAAC------

Mha TACCTTCATCCGAAC------

**ARRC08**

**-35 -10**

App ACCTGAACAATTAAAAGATAACTTAGTTGCGTTATTAGCGGCGTTAAACAAAGCTAAACC

Mva --CTGAACAATTAAAAGATAACTTAAACGCATTATTAGCGGCATTAACTAAAGCTAAGCC

Hdu ------ACAATTAAAAGAAAACCTTCAAGCATTGTTAGCAGCATTAAATAAAGCTAAACC

Asu ------------------------------------------------------------

Mha ------------------------------------------------------------

App GACAACAGCTAAAGGTATCTTCATCAAGAAAGTAAGCATCTCTACAACGATGGGTGCTGG

Mva GACTACAGCGAAAGGTATCTTCATCAAGAAAGTAAGCATCTCTACAACTATGGGTGCTGG

Hdu AACCACTGCGAAAGGTATCTTCATTAAGAAAGTAAGTGTCTCTACAACAATGGGCGCGGG

Asu ------------------------------------------------------------

Mha ------------------------------------------------------------

**sRNA coding sequence**

App TGTTGCTGTTGATCAAGCATCACTTTAATTTCTAATCGAAGTTAAAAC-TTTACAAGGT-

Mva TGTTGCTGTTGATCAAGCTTCACTTTAATTTCTAGTTAGAAGTTAAAACTTTACAGGGTT

Hdu TGTAGCTGTTGAGCAAACCTCACTTTAATACTGACTTTACAGGGT---------------

Asu ------------------------------------------------------------

Mha ------------------------------------------------CTTTACAGGGTC

App CGTGGATTATTGTATAATTTGCGACCTTAACTTGCGAGAGCAAGCTGATGGTGCTTAGCC

Mva GCAGATTATCTGTATAATTTGCGACCTTAACTTGCGAGAGCAAGCTGATGGTGCTTAGCC

Hdu -CGTATTATCGCGTATAATTTCGACCTTAACTTGCGCGAGCAAGCTGATGGTGCTTAGCC

Asu ---------------------------------------------TGATGGTGC-TTGC-

Mha GCAGATTATCTGTATAATTTGCGACCTTAACTTGCGAGAGCAAGCTGATGGTGCTTAGCC

********* * **

App TATCTAAGCCCCGTCCAAGACCGTAGGTGAAT-AAGTTTTCTTATTCTTAATAAAAACCT

Mva TATCTAAGCCCCGTCTAAGACTGTAGGGGTGAAAACT-------------TAATAATCCT

Hdu TATCTAAGCCCCATCCAAGACCGTAGGTGGATAGCTT--GCTATTCTTAATAACAAGCCT

Asu CTATCAAGCCCCGTCCAAGACCGTAGGTGAAATGATGTTAATCATCTCTTAATTTGGCCT

Mha TATCTAAGCCCCGTCTAAGACTGTAGGGGTGAAAACT-------------TAATAATCCT

******* ** ***** ***** * ***

**putative transcription terminator**

App ACGTAGATGGTGAACAGACAGAATTTTCTGCTTCTGGACACCTTAGGCTCAGAAGATTGC

Mva ACATAGATGGTGAACAGACAGAATTATCTGCTTCTGTACACCTTAGGCTCAGAAGATTG-

Hdu ACGTAGATGGTGAACAGACAGAATTATCTGCTTCTGGACACCTTAGCCTCAAGAAGATGC

Asu ACGTAGATGGTGAACAGACAGAATTTTCTGCTTCTGGACACCTTAGGCTCAGAAGATTGC

Mha ACATAGATGGTGAACAGACAGAATTCTCTGCTTCTGGACACCTTAGGCTCAAAGAAGATT

** ********************** ********** ********* ****

App TACTTGCGAT--------------GCAAGCGGTGAGTTTTTTGAGGTTTTTTGTAAATTC

Mva ------CAGCTTATTACAAGCGGTAATATTTTTGGGATTTTTTGTAAATTCTGCTTGAAA

Hdu CG------------TATAAGCGGTAATATTTTTGGGATTTTTTGTAAATTCTGCTCACGA

Asu TACTTGCGAT--------------GCAAGCGGTGAGTTTTTTGAGGTTTTTTGTAAATTC

Mha GCAGTTTGTAGCAATGCAAGCGGTAATATTTTTGGGATTTTTTGTAAATTCTGCTTGAAA

* ** * ***** ** **

App CACTTCGGTGGAGTGTATCAGGAGCTAAAACCA

Mva AGGCAGAGTGTATCAGGA-----GCTAAAACCA

Hdu AAGTGGCAGAGTGTATCAGGAGCTAAAACCA--

Asu CACTTTGGTGGAGTGTATCAGGAGCTAAAACCA

Mha AGGCAGAGTGTATCAGGA-----GCTAAAACCA

**

**ARRC09**

**-35 -10**

App TGGTGTTTAGATGCTAAACGTTTATAAGCACGTTTAATATCGTTCTCACTTGCCCCTTTC

Hin ---TGTTTAGACGCTAAACGTTTATACGCACGTTTAATATCGTTCTCACTCGCCCCTTTT

Asu TGGTGTTTAGATGCTAAACGTTTATAAGCACGTTTAATATCGTTCTCACTCGCCCCTTTC

******** ************** *********************** ********

**sRNA coding strand**

App TGTAAGCCAAGGACTTCGTAGTAATCTTTTTTTGCCATAGTGTTTCGTTTGTAAAATTTT

Hin TTCAATCCAAGGACTTCGTAGTAATCTTTTTTTGCCATAGTGTTTCGTTTGTAAATTTTA

Asu TGTAAGCCAAGGACTTCGTAGTAATCTTTTTTTGCCATAGTGTTTCGTTTGTAAAATTTT

* ** ************************************************* ***

**putative transcription terminator**

App ACGGAAATTTAACCGCTTGTCATGAACTCCCCTCTTTAGCAA------------------

Hin TAGA-AATTTGACCGCTTGTAACATACTCC-------------------------CCTCT

Asu ACGGAAATTTGACCGCCTGTCATAAACTCCCCTCTTTAGCAAAGAGGGGAACTCCCCTCT

* ***** ***** *** * *****

App -------AGAGGGGCAGGGGGAGATTTGGCAGAAGTAACTTTAGCTCATAAGAGAATTTG

Hin TTAACAAAGAGGGGCGGGGGGAGATTTGGCAGAAGTAAATTTAGCTCATAAGAAAATTTG

Asu TTAGCAAAGAGGGGCAGGGGGAGATTTGGCAGAATAGCATTACGCTCATACGAAAATTTG

******** ****************** ** ******* ** ******

App ACATCGTTACCAAATCTCCCCTAACCCCTCTTTTCTAAAGAGGGGGACTTGTT

Hin ATATCGCTATCAAATCTCCCCTAACCCCTCTTTTCTAAAGAGGGGGA------

Asu ACCTTGTCATCAAATCTCCCCTAACCCCTCTTTTCTAAAGAGGGGGAC-----

* * * * *************************************

**ARRC11**

**-35 -10**

App GTTTCAGCAATTGAATATCCTTAAATGATAGGTTTTAGGGGGATAAAAAAATTATTTTTA

Asu -----AAAAT-------------------------------------------TTTTAAA

* * *** *

**sRNA coding sequence**

App TTTTTTTAGAAATTGTCCAATAAATAGGCTTCCCAATTATGTAAAAAGTGCGTATCATAC

Asu TATTTTTAAAAATTGTCGAATAAATAGACTTCCAAAATAGGAAAAAAGTGCGTATCATAC

* ****** ******** ********* ***** ** ** * ******************

**transcription terminator**

App GCACCTCA-AAATCAGCTGAAGCTGACATAGATTGATTAAAGAGTACGGTTTTTTAGCCG

Asu GCACCTCGAAAATCAGGTTAGCCTGAAAGGTTTAAATAAGAAAGTACGGTTTTTTAGCCG

******* ******* * * **** * * ** * * ******************

App TACTTTTTATTTGGATAGTTATCTCGTTAAATTTGTTTTAAATTTAATCACTTACGGAAG

Asu TACTTTTTATTTGGATGTCTCTCTCGTTAAAATCGCTTTAATTTTAATCATTTACGG---

**************** * ********** * * ***** ******** ******

App A

Asu -

**ARRC12**

**-35 -10**

App GTAAGTGACGTTTACGTTTAGTGGTTTTCTTAGTCAAAATATGACGTAAGTGAGATTGTT

Asu GTAAGTGACGTTTACGTTTAGTGGTTTTCTTAGTCAAAATATGACGTAAGTGAGATTGTT

Msc GTAAGTGACGTTTACGTTTAGTTGTTTTCTTAGTCAAAATATGACGTAAGTGAGATTGTT

Hdu -TAAATGACGTTTACGTTTAGTGGTTTTCTTAGTCAAGATATGACGTAAGTGAGATTGTT

Asc -----TGACGTTTACGTTTAGTGGTTTTCTTAGTCAAAATATGACGTAAGTGAGATTGTT

***************** ************** **********************

App TACGTTTGAAACCGCCTGAAGCTGTTTTCTTGAAACGCTTAGCAGCACCACGTACTGTTT

Asu TACGTTTGAAACCGCCTGAAGCTGTTTTCTTGAAACGCTTAGCAGCACCACGTACTGTTT

Msc TACGTTTGAAACCGCCGGAAGCCGTTTTTTTGAAACGCTTCGCAGCACCACGTACTGTTT

Hdu TACGCTTGAAACCGCCTGAAGCTGTTTTCTTAAAACGCTTAGCAGCACCACGTACTGTTT

Asc TACGTTTGAAACCGCCGGAAGCTGTTTTTTTGAAACGCTTAGCAGCACCACGTACTGTTT

**** *********** ***** ***** ** ******** *******************

**sRNA coding sequence**

App TAATTTTAGGCATTGTTTTATAAACTCCGCATTGTTTTATCTAATACATAATAATCAGGC

Asu TAATTTTAGGCATTGTTTTATAAACTCCGCATTGTTTTACATAATACACAATAATTAGGC

Msc TAATTTTAGGCATTGTTAAAAAAACTCCGCATTGTTTTGTTTGATACACAATAATCAGGC

Hdu TAATTTTAGGCATTGTTTTAGAAACTCCGCATTGTTTGTTAATCAAAAGTCAATTA-GGC

Asc TAATTTTAGGCATTGTTTAAAAACTCCGCATTGTTT--TGTTAACACTTGATAGTCAGGC

***************** * ** * * ** * * * ***

App GAAAAATACGCTTATCGCTAACCGTCTTTTTACTTGC--AAAGCACTAATTATCTCGAAC

Asu GAAAAATGCGATTATTGCTAATCATATTTTTACTTGG--AAAGCGCTAATTATCTCGAA-

Msc GAAAAATACCGCCATTTCTGACCGCACTTTTACTTGG--AAAGCACTAATTATCTCTGAT

Hdu GAACAGTATTACTAATGTTACTTGGAAGCAACT------AAT-GACATATAAAAAATAGT

Asc GAAAAACACGATTATTTTCAACCGCACTTTTGCTTTTGTAAAGCACTGCTTATCTCTCAA

*** * * ** * * *

App AGAGCGTTACCGCTCATAGCCCTTTACGGCTAAAAAGCAAATCAGGCTGCGAAATGTGCC

Asu AGAGCGTTACCGCTCATAAGCTTTGCAGCTTAAAAAGCAAATCAGGCTGCGAAATGTGCC

Msc TGAGCGTTACCGCCCATAAGCCTTTTACGGCAAAAAGCAAATCAGGCTGCGAAATGTGCC

Hdu TTCCACTATCTAACTTATTTAACATAAGTATAAAAAGCAAATCAGGCTGCTGAGTGTGCC

Asc ATGGCATT--------------ACTGCCGTGAAAAAGCGAATCAGGCTGCGAAATTTACC

* ******* *********** * * * **

**transcription terminator**

App TGTAGATTGCTTTTGTTTCTCTTTTTTACAAAAGAGAAAACGGACGGATTTTATAAGATC

Asu TGTAGATCGCTTTTGTTTCTCTTTTTTACGAAAGAGAAAACGGACGGATTTTATAGGATC

Msc TGTAGATTGCTTTTGTTTCTCTTTAAT--AAAAGAGAAAACGGACGGATTCTATAGGATC

Hdu TGTAGATTACTTTATTTCT-GTTATTTATATAACAAGAAACGGGCGAATTTTATAAGATC

Asc TGTGGATCACTTTTGTTTTCCGA--------TATGAAAAACGGACGAATTTTATAGGATT

*** *** **** ** * ****** ** *** **** ***

App CGCCCGTTTTATGCAAGAATAAA

Asu CGCCCGTTTTATGCAAGAATAAA

Msc CGCCCGTTTTATGCAAGAATAAA

Hdu CGCCCATTTTATGCAAGAATAAA

Asc CGTCCGTTTTATGCAAG------

** ** ***********

**ARRC14**

**-35 -10**

App ATGCAATGTTTTGTCGGAAAATCGTTTGCTTGTAGTGAAAAATGCTATTGACTTCACTCT

Asu -----------------GAAAACGTTTGCGAGGAGTGAAAAATTCTATTGACTTCACTCT

*** ******* * ********** ****************

**sRNA coding sequence**

App ATTTGAAGTTAATATGTACCCGTATT-TCAAATAACTATGCAA--ACATAAACACACACA

Asu ATTTGAGGCTAATATAGCTCTTGTATTTCGAATAACTATGCAAACATAAACACACACACA

****** * ****** * * ** ************* * * * *********

**putative** **transcription terminator**

App ACATAATGGAAAAACGACTATCTCTTCGACTGCTTCTGTTATCGCATTTCGTGCGAGGAT

Asu ACACAATGGAAAAACTACTTTCTCTCCGACTTCTTCTGTTATCGCATTCTGTGCGAGGAT

*** *********** *** ***** ***** **************** **********

App AAAGGTCGTTCGGAGTGAAAGCGTTCACTATCCACACTTTGCCCGCACATTGATGCGGGT

Asu AAAGGTTGGTTGGACGAAAATAGTTCACTATCCACACTTTACCCGCACATCGATGCGGGT

****** * * *** *** ****************** ********* *********

App TTTTTTTACTTATA

Asu TTTTTTTA------

********

**ARRC16**

**-35 -10**

App GCTAATGTCGTTTCAATAGATTGAGCCA--TTTATGATTCCTTATATCTTGTCTTAAATA

Asu GCTAATGTTGCTTCAATAGATTGAGCCATTTGCTTGATTCCTTATATCTTGTTTTAAATA

Hdu GCTAATATTGCTTCAATAGATTGAGCCATTTGTATTC-------CTTTTCTGATTAACAT

Mha -----------TTCGATAGCTTGAGTCATTTATCTTC-------CTTTTAGTCTTGAATA

Mva -----------TTCGATAGATTGAGTCATTGATCTTC-------CTTCTCTT--TTATCA

*** **** ***** ** * * * * *

**sRNA coding sequence**

App TGAAAATAACTTCTATTATAACGGGATACATTGGAATTGATAAGGTTTAATCGGCAATAG

Asu TGAAAATAGAATCTATTATAACGAGATACGTTGTGATTGATAAGGCTTAATCGGCAATAG

Hdu ATAAAATGTGGTCTATTATAACGGGCTGAATTGAAATTGATAGCGCTAAAAAGGCGATAG

Mha TAAAAAATGGATCTATTATAGCGGTATGAATTGAAATT----------GATAGGCTATAG

Mva ATAAAAATGGCTCTATTATAGCGGTATGAATTGAAATTAATAGAGGTTTTGGCGGA--TA

**** ********* ** * *** *** *

**putative transcription terminator**

App ACGAAAAAAAACGCCTCATTTTGTGGGGGAATGAGGCGTAA-GAGTTGGAGTGATTACCT

Asu ACGAAAAAAAACGCCTCATTTTGTGGGGGAATGAGGCGTAA-GAGTTGGAGTGATTACCT

Hdu GCAAAAAAAAACGCCTCATTTTGTGGGGGAATGAGGCGTAAAGAGTTGGAGTGATTACCT

Mha GCGAAAAAAAACGCCGTATTTTGTGGGGGAATACGGCGTAAAGAGTTGGAGTGATTACCT

Mva AGCAAAAAAAACGCCGTATTTTGTGGGGGAATACG-------------------------

************ *************** *

App ATTATTTTTGAAGTGAGTTAATGATAATAATTCTCATCTAGTTAAGCAATAGTCTTTACA

Asu ATTATTTTTGAAGTGAGTTAATGATAATAATTCTCAAGTAATTAAGCAAGAGGCTTTACA

Hdu ATTATTTTTGAATTGAGTTAATGATAATGATTCTCAATGCATTAAGCAAATAAATTTACA

Mha ATTATTTTTGAAGTGATTTAATGA-----------------------------TAATGAT

Mva -GCGTAAAGAGTTGGAGTGATTACCTATTATTTTTGAAGTGAGTTGATGATAATA-----

* ** * * *

App ATTTCTTACAAATGAGAAT-

Asu AATTCTTACAAATGAGAAT-

Hdu AAAACTTACAAATAAGAAT-

Mha TATCATCTGTTAAGTCAATA

Mva -----GTTCTCA--------

**ARRC17**

**-35 -10**

App TCGTGCTTCAGTTAAGAAATTATGCCGTAACTGTAAAGTTGTTAAACGTCAAGGTGTTGT

Asu TCGTGCTTCAGTTAAGAAATTATGCCGTAACTGTAAAGTTGTTAAACGTGAAGGTGTTGT

Hdu TCGTGCTTCAGTTAAGAAATTATGCCGTAACTGTAAAGTTGTTAAACGTGAAGGTGTTGT

Mha TCGTGCTTCAGTAAAAAGAATGTGCCGTAATTGTAAAGTAATCAAACGTGAAGGTGTGGT

Mva TCGTGCTTCAGTTAAAAGAATGTGCCGTAATTGTAAAGTAATCAAGCGTGAAGGTGTGGT

Hps TCGTGCTTCAGTTAAAAGAATGTGTCGTAACTGCAAAGTTGTTAAACGTGAAGGTGTTGT

Hin TCGTGCTTCCGTAAAGAAAATGTGTCGTAACTGTAAGATTGTTAAACGTGAGGGTGTTGT

Hsm TCGTGCTTCAGTCAAGAAAATGTGTCGTAACTGTAAAATTGTTAAACGTGAAGGTGTTGT

Gan TCGTGCGTCCGTAAAGAAATTATGTCGTAACTGTAAGATTGTTAAACGTGAAGGCGTAGT

Asc TCGTGCTTCCGTAAAAAGAATCTGTCGTAACTGTAAAATTGTTAAACGTGAAGGTGTCGT

****** ** ** ** * * * ** ***** ** ** * * ** *** * ** ** **

**sRNA coding sequence**

App TCGCGTAATTTGTAGCGATCCTAAACACAAACAACGTCAAGGTTAATT-AGTATTCTTTC

Asu TCGCGTAATTTGTAGCGATCCTAAACACAAACAACGTCAAGGTTAATT-AGTATTCTTTC

Hdu TCGTGTAATTTGCACCGATCCTAAACATAAGCAACGTCAAGGTTAATTAAGTATTCTTTC

Mha TCGTGTAATTTGTAGCGATCCTAAACACAAACAACGTCAAGGTTAATTTACGCATATTTC

Mva TCGTGTAATTTGTAGCGACCCTAAACACAAACAACGTCAAGGTTAATTTACGCATATTTC

Hps ACGCGTAATTTGTAGCGACCCTAAACATAAACAACGTCAAGGTTAATT-GACATTATTTC

Hin ACGCGTATTGTGTAGCGACCCTAAACACAAACAACGTCAAGGTTAATTAACATTT--TTC

Hsm TCGTGTATTATGTAGCGACCCTAAGCATAAACAGCGTCAAGGTTAATTGATATTT--TTC

Gan TCGCGTGCTTTGCAGCGATCCTAAACATAAACAACGTCAAGGTTAATTGATATATTTTTC

Asc ACGTGTTTTATGTACCGACCCTAAACATAAACAACGTCAAGGTTAATTGATAAAT--ATC

** ** * ** * *** ***** ** ** ** ************** * **

App TTGCAAAGAACCCGCTGAGCAGGTATACTGCTCAGCTCAT---TCGTCC----TGATATG

Asu TTGCAAAGAACCCGCTGAGCAGGTATACTGCTCAGCTCAT---TCGTCC----TGATATG

Hdu TTGCAAAGAACAAGCTGAGCAGGTATACTGCTCAGCTAAT---TTGTCC----TGATATA

Mha TTGCAAAGAACCCGCTGAGCAGTTATACTGCTCAGCTCAT---TCGTCC----TGATATA

Mva TTGCAAAGAACCCGCTGAGCAGTTATACTGCTCAGCTCAT---TCGTCC----TGATATA

Hps TTGCAAAGAACCTGCTGAGCAGTTATACTGCTCAGCTCATT--TCGTCC----TGATATG

Hin TTGCCAAGAACCAGTTGAGTAGTTATACTGCTCAACTCATTTATGTCCT----TGATATT

Hsm TTGCAAAGAACCAGCTGGGTATATATAATACTCAGCTCATTTATGTCCT----TGATATG

Gan TTGAAAAGAATCGGTTGAGTGGGTATACTGCTCAACTCTTTTGTATAGTGTATTGGTATG

Asc TTGCAAAGAACAGGTTGAGCAGTTATACTGCTCAGCTCATTTATATCCT----TGGCATA

*** ***** * ** * **** * **** ** * * ** **

**putative** **transcription terminator**

App CTGTTTGAGTATCCTGAAACGGGCTTTTCAAGATCAGCATATCAATAAACTTAAATAATA

Asu CTGTTTGAGTATCCTGAAACGGGCTTTTCAAGATCAGCATATCAATAAACTTAAATAATA

Hdu CTGTTTGAGTATCCTGAAACGGGCTTTTCAAGATCAGTATATCATAAACTT-AAATAATA

Mha CTGTTTGAGTATCCTGAAACGGGCTTTTCAAGATCAGTATATCAATAAACTTAAATAATA

Mva CTGTTTGAGTATCCTGAAACGGGCTTTTCAAGATCAGTATATCAATAAACTTAAATAATA

Hps CTGTTTGAGTATCCTGAAACGGGCTTTTCAAGATCAGCATATCAATAACTTTAGTTAAAT

Hin CTGTTTGAGTATCCTGAAAACGGGCTTTTCAAGATCAGAA----TATCAAATTAATTAAA

Hsm CTGTTTGAGTATCCTGAAACGGGCTTTTCAAGATCAGTATATCAAATTTAATCAATAAAA

Gan TTGTTTGAGTATCCTGAAACGGGCTTTTCAGATCAACA-TACCAGATTAGTTAAATAATA

Asc CTGTTTGAGTATCCTGAAACGGGCTTTTCAAGATCAGTATGTCA-----AATTAGTTAAA

****************** ** *** * *

App GGAGTGC-ATA----

Asu GGAGTGC-ATA----

Hdu GGAGTGC-ATA----

Mha GGAGTGCATA-----

Mva GGAGTGCATA-----

Hps AGGAGTGCATA----

Hin ATATAGGAGTGCATA

Hsm TAATAGGAGTGCATA

Gan G-----GAGTGCATA

Asc ATATAGGAGTGCATA

**ARRC18**

**-35 -10**

App TTCCAATATATTGATAAACCTCGTAGTAAATGGAACTATAAAAAGAATGACGAACTCTTA

Asu TTCCAATATATTGATAAACCTCGTAGTAAATGGAACTATAAAAAGAATGACGAACTCTTA

************************************************************

**sRNA coding sequence**

App AAATAGTTTCCTTTTAACTGCTACGATGAGTCGCAAATTCCCGATAATTTCGCCAATTTT

Asu AAATAGTTTCCTTTTAACTGCTACGATGAGTCGCAAATTCCCGATAAATTCGCTAATTTT

*********************************************** ***** ******

App GCAAGATTTTATTCGCAAAATTGGCGAAATTGGAGTAATCTAGGCAAGATTTTTACTCTG

Asu GCAAGATTTTATTCGCAAAATTGGCGAATTTGGAGTAATCTAGGCAAGATTTTTACTCTG

**************************** *******************************

**putative transcription terminator**

App CATAATGTTGTGCGGAGTTTCTCTTTTTTAATTGACTATAAAAACGGAATCTCA

Asu CATAATGTTGTGCGGAGTTTCTCTTTTTTAATTGGCTATAAAAACGGAATCTCA

********************************** *******************

**ARRC20***

App -----------------ATAAAAAGTTAAAGTTCAAGAATTGCAAAGAATTGACAAGTTA

Asu TAAAATTCAAGAATTGCAAAGAATTGACAAGTTAGGTTATATGGTTTGA----------C

Mva ------------------------------------------------------------

Mha ------------------------------------------------------------

App GGTTATAGCATTTGACGCTAAAACGGTTTAGCGATATTATTTTTTCGTATTGTTCCTCCT

Asu GCTAAAACGGTTTAGCGATATTATTTT--------------TTTCCGAAATGTTCCTCCT

Mva ---------ATTTGACGGCTAAAACGTTTTAGCCTATTATGCACATCGTTAATTCCTCCT

Mha ---------ATTTGACGGCTAAAACGTTTTAGCCTATTATTTACACCGTTAATTCCTCCT

*** ** * * ********

App TAGTTCAGTCGGTAGAACGGTGGACTGTTAATCCATATGTCGCAGGTTCGAGTCCCGCAG

Asu TAGTTCAGTCGGTAGAACGGTGGACTGTTAATCCATATGTCGCAGGTTCGAGTCCCGCAG

Mva TAGTTCAGTCGGTAGAACGGTGGACTGTTAATCCATATGTCGCAGGTTCGAGTCCCGCAG

Mha TAGTTCAGTCGGTAGAACGGTGGACTGTTAATCCATATGTCGCAGGTTCGAGTCCCGCAG

************************************************************

App GAGGAGCCA-CTAATTTCCTTTAGTTTTGCTTTTGTTCTTGTTTTATTTGTCTCCTTTTA

Asu GAGGAGCCAACTAATTTCCTTTAGTTTTGCTTTTGTTCTTGTTTTATTTGTCTCCTTTTA

Mva GAGGAGCCAAAGATTTTT-----TATTTTCTTTTGTTCTTGTTTTATTTGTCTCCTTTTA

Mha GAGGAGCCAAGATTTTA-------TTTTTCTTTTGTTCTTGTTTTATTTGTCTCCTTTTA

********* ** *** *******************************

**putative transcription terminator**

App TAAAACAGTGATTCATACCTCCAGAATTAGAAAAAAAGAAACC---CCGTAGGTTTTCTA

Asu TAAAACAGTGATTCATACCTCCAGAATTAGAACAAAAGAATTTGCCCCGTAGGTTTTCTA

Mva TAAAACAGTAATTCAAACCTCCAGAATTAGAACAAAAG----------------------

Mha TAAAACAGTAATTCAAACCTCCAGAATTAGAACAAAAG----------------------

********* ***** **************** *****

App CGGGGTTTC

Asu CGGGGTTTC

Mva ---------

Mha ---------

*No promoter region was searched for this sRNA, because RtTs are known as small transcripts released from specific tRNAs molecules, with regulatory roles.

**ARRC21***

App AAAAAGCACTTGACCATTTTGTTTAAATCCGTATTATATGCGCCTGTTACGCAACGTTAA

Asu AAAAAGCACTTGACCATTTTTTTTAAATCCGTATTATATGCGCCTGTTACGCAACGTTAA

Hdu AAAAAGCACTTGACGCTCTTACTAAAATCCGTATTATACACGCCTGTTATGCAATATTAA

Hps ------------------------AAATCCGTATGATACGCCCTCGTTACGCAACGTTAA

Mva ------------------------AAATCCGTATGATATGCCCTCGTTACGCAAGATTAT

Mha ------------------------AAATCCGTAAGATATGCCCTCGTTACGCAAGATTAT

Agp ----------------------AAATCAGTATTATAAGCGCTCGTTGTTAAATGTTACTT

Gan ------------------------------------------------------------

Hsm -------------------------GTAATA------TATCCGCTCGTTACAAACAATGA

Agc ----------------------AAATCAGTATTATAAGCCCTCGTTGTTAGATGTT-AAC

Asc ------------------------------------------------------------

Msc ------AAATCC-------------GTATTATAAGCACCCGTTACACAGCGTAACCTTGT

Pmu ------------AAATCAGTAT------TATAAGCCTCCGTTACGCAATGATATGCAAAT

App GGGTCGTTAGCTCAGTCGGTAGAGCAGCGGACTTTTAATCCGTTGGTCGAAGGTTCGAAT

Asu GGGTCGTTAGCTCAGTCGGTAGAGCAGCGGACTTTTAATCCGTTGGTCGAAGGTTCGAAT

Hdu GGGTCGTTAGCTCAGTCGGTAGAGCAGCGGACTTTTAATCCGTTGGTCGAAGGTTCGAAT

Hps GGGTCGTTAGCTCAGTCGGTAGAGCAGCGGACTTTTAATCCGTTGGTCGAAGGTTCGAAT

Mva GGGTCATTAGCTCAGTCGGTAGAGCAGCGGACTTTTAATCCGTTGGTCGAAGGTTCGAAT

Mha GGGTCATTAGCTCAGCCGGTAGAGCAGCGGACTTTTAATCCGTTGGTCGAAGGTTCGAAT

Agp GGGTCGTTAGCTCAGTCGGTAGAGCAGCGGACTTTTAATCCGTTGGTCGAAGGTTCGAAT

Gan GGGTCGTTAGCTCAGTCGGTAGAGCAGCGGACTTTTAATCCGTTGGTCGAAGGTTCGAAT

Hsm GGGTCGTTAGCTCAGTCGGTAGAGCAGCGGACTTTTAATCCGTTGGTCGAAGGTTCGAAT

Agc GGGTCGTTAGCTCAGCCGGTAGAGCAGCGGACTTTTAATCCGTTGGTCGAAGGTTCGAAT

Asc GGGTCGTTAGCTCAGTCGGTAGAGCAGCGGACTTTTAATCCGTTGGTCGAAGGTTCGAAT

Msc GGGTCGTTAGCTCAGTCGGTAGAGCAGCGGACTTTTAATCCGTTGGTCGAAGGT------

Pmu GGGTCGTTAGCTCAGTCGGTAGAGCAGCGGACTTTTAATCCGTTGGTCGAAGGTTCGAAT

***** ********* **************************************

**putative transcription terminator**

App CCTTCACGACCCACCATTTAACTTGCAACGCCTTCTAAAGGGTCGTTA-GC------TCA

Asu CCTTCACGACCCACCATTTAACTTGCAACACCTTCTAAAGGGTCGTTA-GC------TCA

Hdu CCTTCACGACCCACCACTTAATAATTTGCACACCTACATTCTAAAGGG-TCGTTAGCTCA

Hps CCTTCACGACCCACCACTTAATCAGCGTTCTTTTCTAAAGGGTCGTTA-GC------TCA

Mva CCTTCATGACCCACCATTTAATCTTCAAAACACCTCTTTGGGTCATTA-GC------TCA

Mha CCTTCATGACCCA-CCATTAATCTTCAAAACACCTCTAAGGGTCATTA-GC------TCA

Agp CCTTCACGACCCACCAATTTAACAATATACCCCTTCAAAGGGTCGTTAG-------CTCA

Gan CCTTCACGACCCACCATTTATTATAATACCCTTATGGGTCGTTAGCTC-AGTCGGTAGAG

Hsm CCTTCACGACCC----ACCATTTTATTTGTAATATCCCAATTTATGGG-TCGTTAGCTCA

Agc CCTTCACGACCCACCACTTTTAAACAATA----TATCCCAAATAATGGGTCGTTAGCTCA

Asc CCTTCACGACCCACCACTTAAATCTGGTTTACCCATATCAGTATGGGT-CGTTAGC-TCA

Msc --------------------TCGAATCCTTCACGACCCACCACTTAAA-ATTTGGT-TTT

Pmu CCTTCACGACCCACCACTTAA---------------------------------------

App GTCGGTAGAGCAG-----------------------------

Asu GTCGGTAGAGCAG-----------------------------

Hdu GTCGGTAGAGCAG-----------------------------

Hps GTCGGTAGAGCAG-----------------------------

Mva GTCGGTAGAGCAG-----------------------------

Mha GTCGGTAGAGCAG-----------------------------

Agp GTCGGTAGAGCAG-----------------------------

Gan C----------AG-----------------------------

Hsm GTCGGTAGAGCAG-----------------------------

Agc GTCGGTAGAGCAG-----------------------------

Asc GTCGGTAGAGCAG-----------------------------

Msc ATCCATATTCAATATGGGTCGTTAGCTCAGTCGGTAGAGCAG

Pmu ------------------------------------------

*No promoter region was searched for this sRNA, because RtTs are known as small transcripts released from specific tRNAs molecules, with regulatory roles.
